# Libraries as Spaces for Inclusive Communities: A Literature Review

**DOI:** 10.12688/f1000research.175122.1

**Published:** 2026-01-09

**Authors:** Salma Paola Rodríguez Poveda, Ana Dolores Vargas Sánchez, Diego Efrén Rodríguez Cárdenas, Cesar Augusto Niño Hernández

**Affiliations:** 1 Universidad de La Sabana, Chia, Cundinamarca, Colombia

**Keywords:** Library, Inclusivity, Community, knowledge, Education

## Abstract

Recognizing and appreciating the diversity of people around the world is a necessary step in achieving equality for all. The opening of equitable chances for society as a whole is one of the ways in which libraries contribute to the reduction of the exclusion gap. Despite their expanding relevance, institutions like libraries demonstrate inconsistent patterns of integration and research. This literature analysis aimed to examine the methods employed by public libraries to foster social inclusion across various populations from 2014 to 2024. A comprehensive literature review was performed, identifying n = 69 interventions that fulfilled the inclusion criteria according to PRISMA methodological principles. The findings indicate a necessity for collaboration between libraries and other organizations to enhance their effectiveness in promoting social inclusion. The findings underscore the necessity of adopting a comprehensive perspective on inclusion in libraries, which encompasses an examination of infrastructure and participation in physical spaces, as well as additional aspects and dimensions, integrating both physical and digital inclusion.

## 1. Introduction

Today, inclusion in the globe is a concept and a social change trend that is widely seen from various viewpoints, since it entails recognising and appreciating human uniqueness and pursuing equal chances for everyone. Libraries, for their part, provide open and free access to information and culture for the entire community, regardless of color, gender, age, social or economic background, or educational level, among other things. Libraries can now assist close the exclusion gap by providing equal access to information.

Ainscow ^1^ states that for thirty years, numerous initiatives have been undertaken to advance inclusion in educational settings, typically according to a philosophy known as education for all. To attain this objective, it is essential to not only enhance schools but also to include the entire community. In this context, public libraries, which engage in non-academic educational activities, serve as an appropriate venue for fostering inclusivity.

Inclusion has been characterized in various ways. Pionke et al. ^2^ state that it involves the act of establishing secure environments or spaces where individuals feel included, appreciated, and respected. In this context, when discussing inclusion, we are referring to an environment that is accessible to all individuals, free from any form of discrimination. Libraries, in particular, serve as environments that foster such opportunities, possessing both the personnel and the space necessary to facilitate inclusive experiences. Furthermore, libraries were established with the intent of serving as repositories of knowledge. Their role is to facilitate information retrieval for individuals and to ensure that these services are accessible to all (Milano, 2013 cited in ^3^).

In the past decade, research has examined how libraries have approached inclusion from several viewpoints, including digital literacy and accessibility for individuals with impairments. The ethical and social function of librarians as facilitators of social inclusion has been examined. ^3^ Due to swift technology advancement, libraries have had to adjust, enabling them to restructure for enhanced inclusivity in all dimensions.

Alcívar et. al. ^4^ contend that the Marrakesh Treaty (2013) facilitates access to books and other physical materials in libraries for individuals who, due to visual or physical disabilities, are unable to utilize them in their original version. The treaty permits the alteration of copyright laws to transcribe materials into Braille or other formats, so enhancing access to information and knowledge for individuals with impairments.

Consequently, inclusion has emerged as a prominent societal trend, with libraries, as public and cultural institutions, asserting their role as essential venues for fostering inclusion among diverse communities. This is why it is regarded as a crucial area of inquiry and examination, despite the plethora of published studies, in which concerns persist regarding how libraries have evolved into transforming environments. Prihatin et al. ^5^ assert that rural libraries serve as venues and catalysts for social transformation via inclusive programs. These venues enable the surrounding communities of villages or towns to foster integration and cultivate genuine inclusion among participants of these meetings.

This article aims to examine the scientific data concerning the role of public libraries as environments that foster social inclusion. This study aims to address the research question: How have public libraries executed strategies and initiatives to foster social inclusion?

Thus, in a second stage, we strive to examine the strategy adopted by theorists and their varied contributions to the subject, seeking to learn from the literature the structured approach to controlling inclusion procedures from libraries. It is envisaged that this review will provide a critical and intelligible synthesis that will contribute to academic and research domains. It is also intended that it would help with future studies, where it will be possible to look at ways to make things better and how libraries help communities.

## 2. Method

This study was generated using qualitative data analysis, wherein the existing literature on the role of libraries as hubs of community inclusion globally was critically assessed and synthesized. The analysis of the information involved considering emergent categories from the data review, utilizing a qualitative approach centered on synthesis and critical evaluation of the existing evidence on the specific topic discussed in the literature. ^6^

### 2.1 Search criteria

The search covered the years 2014-2024. This decade was chosen for the review because it is when inclusion began to infiltrate communal settings more strongly, shifting from legal or theoretical methods to practical applications relevant to modern society. “But it is no longer enough to adapt to this new digital landscape; it is now essential to make a shift both conceptually and in practice” ^7^ (p. 105). It also looked at how, throughout this period, libraries played a role in community inclusion, becoming what some authors referred to as “social capital partners” ^7^ (p. 103).

The inquiry was performed utilizing the SCOPUS database. This database was chosen due to its status as one of the most comprehensive sources, recognized for the accuracy and dependability of its data. ^8^ The review encompassed articles written in both Spanish and English, while excluding books, book chapters, conference papers, and meta-analyses.

The study concentrated on libraries broadly, and upon reviewing the articles, after excluding those based on language and document type, 200 articles pertinent to the guiding issue were first identified; however, on June 3, 2025, only 198 papers remained without any further exclusions applied.

### 2.2 Source identification

The research was executed in compliance with the PRISMA statement (Preferred Reporting Items for Systematic Reviews and Meta-Analyses). A database search was conducted utilizing the following concatenated keywords: Library AND Inclusive Communities AND Initiative, to encompass all relevant facets of this review issue and enable the incorporation of diverse studies.

The following search equation was entered into the database SCOPUS: (TITLE-ABS-KEY (“library” OR “libraries”) AND TITLE-ABS-KEY (“inclusive communities” OR “social inclusion” OR “diverse communities” OR “access to information” OR “community engagement”) AND TITLE-ABS-KEY (“program*” OR “initiative*” OR “strategy” OR “services” OR “education”)) AND PUBYEAR > 2013 AND PUBYEAR < 2025 AND PUBYEAR > 2013 AND PUBYEAR < 2025 AND (LIMIT-TO (SUBJAREA,“ARTS”) OR LIMIT-TO (SUBJAREA,“DECI”) OR LIMIT-TO (SUBJAREA,“SOCI”) OR LIMIT-TO (SUBJAREA,“PSYC”)) AND (LIMIT-TO (DOCTYPE,“ar”)) AND (LIMIT-TO (EXACTKEYWORD,“Libraries”) OR LIMIT-TO (EXACTKEYWORD,“Library”) OR LIMIT-TO (EXACTKEYWORD,“Academic Libraries”) OR LIMIT-TO (EXACTKEYWORD,“Library Services”) OR LIMIT-TO (EXACTKEYWORD,“Accessibility”) OR LIMIT-TO (EXACTKEYWORD,“Inclusion”) OR LIMIT-TO (EXACTKEYWORD,“Sustainable Development Goals”) OR LIMIT-TO (EXACTKEYWORD,“Community Development”) OR LIMIT-TO (EXACTKEYWORD,“Community-Institutional Relations”) OR LIMIT-TO (EXACTKEYWORD,“Rural Libraries”) OR LIMIT-TO (EXACTKEYWORD,“Libraries, Digital”) OR LIMIT-TO (EXACTKEYWORD,“Library Associations”) OR LIMIT-TO (EXACTKEYWORD,“Equity”) OR LIMIT-TO (EXACTKEYWORD,“Academic Library”)) AND (LIMIT-TO (LANGUAGE,“English”) OR LIMIT-TO (LANGUAGE,“Spanish”). The suggested inclusion criteria included publication dates between 2014 and 2024, English or Spanish language, and only finished works from the following theme areas: Social Science, Arts and Humanities, Decision Sciences, and Psychology. Libraries, accessibility, inclusion, community development, equity, rural libraries, and digital libraries were some of the keywords used.

### 2.3 Data extraction and analysis

The preliminary search produced 2,120 items. Subsequent to the initial search, exclusion criteria were defined: title, absence of open access, and insufficient relevance to the research during the abstract evaluation, yielding 198 articles.

**Figure 1.**
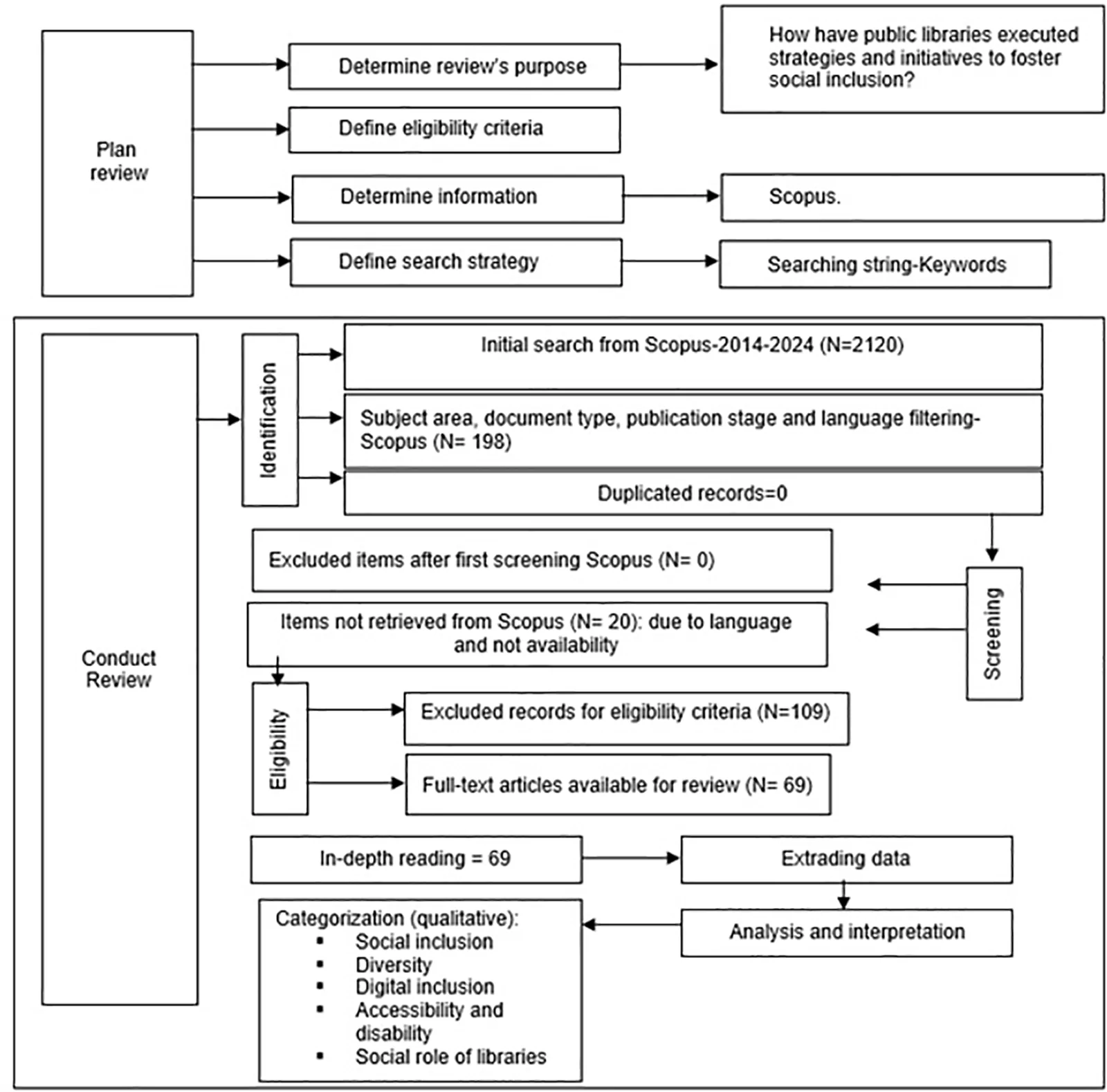
Detailed diagram of information source identification. Source: PRISMA (2020) flow diagram. Prepared by the authors.

Following the elimination of texts that failed to satisfy the inclusion criteria, a total of 69 articles were incorporated into the qualitative analysis. The content analysis included initial reading, coding, delineation of emerging categories, and ultimately, the description and systematization of the data (see Figure 1).

Each article was thoroughly reviewed in its entirety, and recurring patterns were identified through open coding. The preliminary stage of categorization was employed to develop analytical categories and subcategories. This classification facilitated a chronological and thematic analysis to discern patterns, overlaps, and deficiencies. Given the descriptive nature of the review, a formal assessment of risk of bias or methodological quality (e.g., utilizing CASP, AMSTAR, or ROBIS) was not conducted. Therefore, the conclusions derived from the findings should be understood within the scope of the analysis, without relying on categorical generalizations.

The results were systematically categorized thematically and displayed with graphs and heat maps generated using Visme.net and data aggregated in Excel, facilitating the illustration of temporal and geographical trends. All quantitative meta-analyses were eliminated, and aggregate measures of effect and heterogeneity were not calculated, as they beyond the descriptive scope of the study. Publication bias was not addressed; nevertheless, its potential influence is recognized in the limits section. This technique provides a critical qualitative synthesis that amalgamates diverse data, facilitating a greater comprehension of prevalent themes related to inclusion in libraries.

## 3. Results

**Figure 2.**
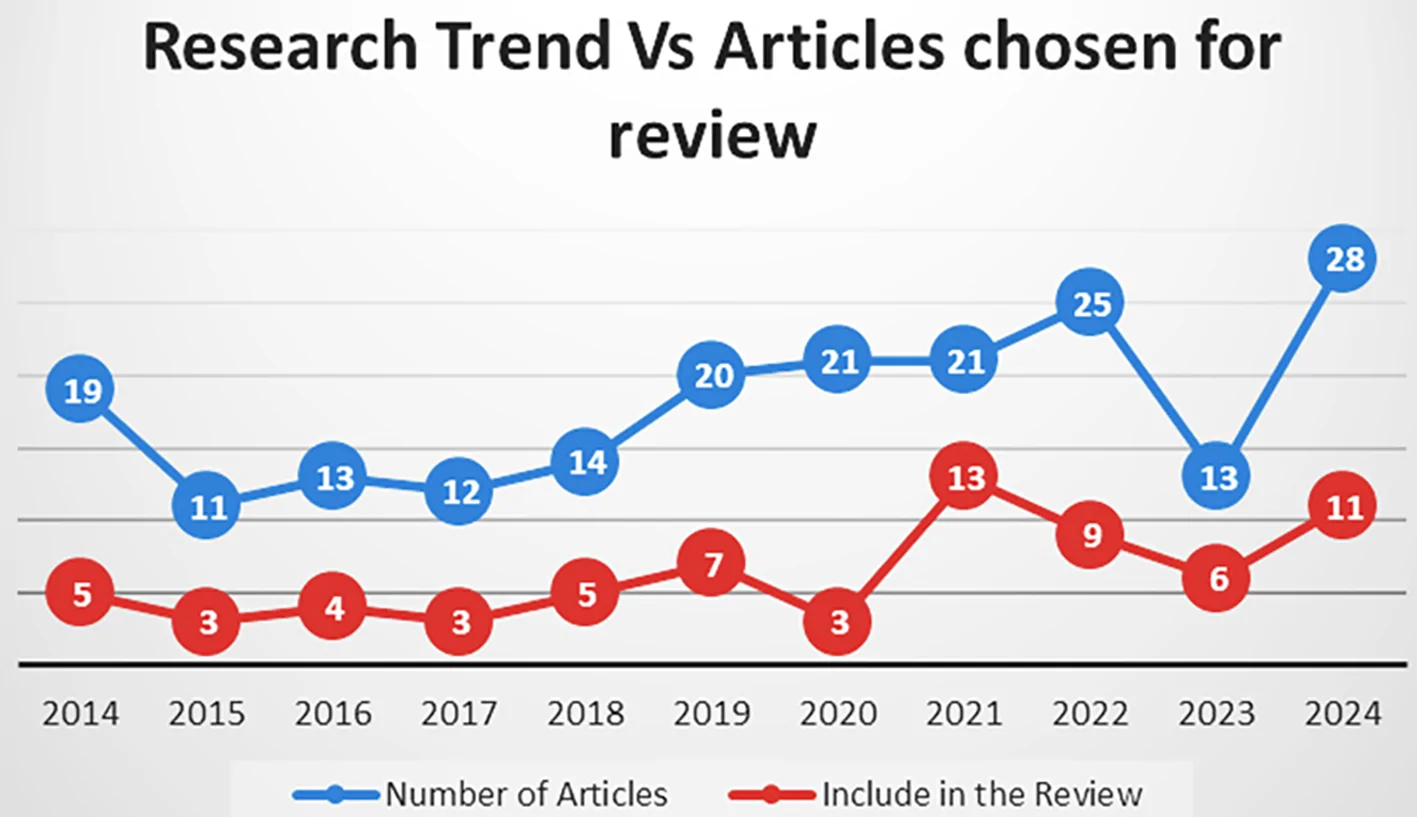
Examination of the research sample. Source: Original composition by the authors.

Scholarly output about the enhancement of inclusion in libraries has escalated over the past decade, as evidenced by the time series derived from the documentary corpus. A statistical computation was conducted to validate the number of studies reviewed, yielding a dependability rate of 85% (see Figure 2).

In the year 2014, there was a significant amount of study conducted on libraries as centers for social inclusion. In comparison to 2014, the amount of literature produced on this subject decreased between the years 2015 and 2019, yet it stayed relatively unchanged overall. Between the years 2020 and 2022, there was a spike in the production of literary works, which culminated in a maximum of 25 articles in the year 2022. A significant decrease in the amount of literary work produced took place in the year 2023, with the number of pieces falling from 25 to just 13. Twenty-eight pieces were published in 2024, all of which addressed social inclusion from a variety of angles and examined community participation in libraries. This was the year that literary creation achieved its highest level in the previous ten years.

### 3.1 Geographic distribution

**Figure 3.**
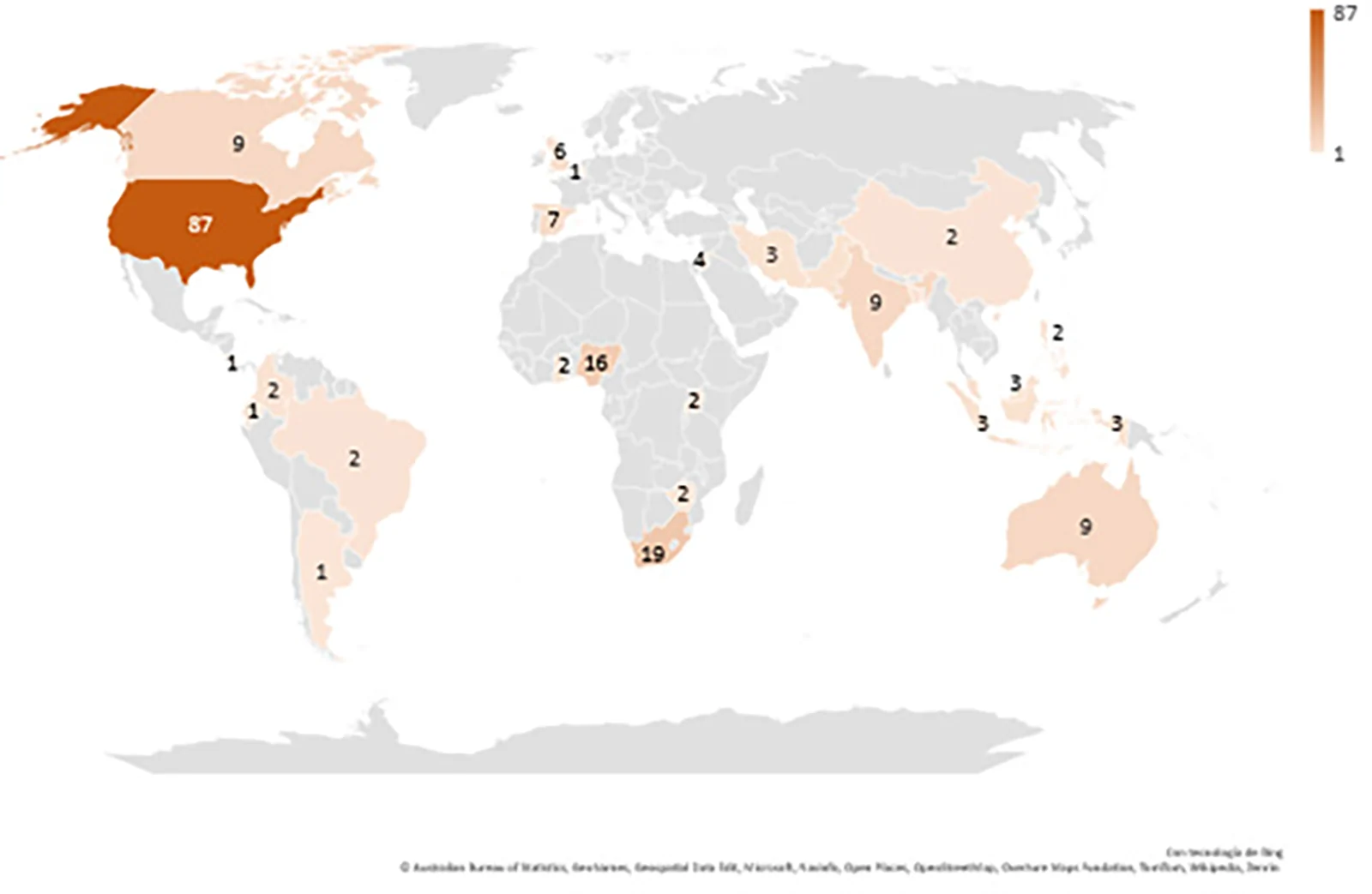
Heatmap of the inquiries. Source: Original composition by the authors.

Figure 3 illustrates a heat map depicting the quantity of papers published by country. The research analyzed were not omitted based on nation but were filtered by language, including only articles in English and Spanish. The research indicates that the scholarly emphasis on libraries’ role in promoting social inclusion is predominantly situated in the United States (87), South Africa (19), Nigeria (16), India (9), Canada (9), and Australia (9), collectively representing 75% of the published papers. Countries including Spain (7), the United Kingdom (6), Jordan (4), Indonesia (3), Iran (3), Malaysia (3), Pakistan (3), China (2), Bangladesh (2), Brazil (2), Colombia (2), Ghana (2), the Philippines (2), Uganda (2), Zimbabwe (2), Argentina (1), Belgium (1), Costa Rica (1), and Ecuador (1) have made substantial contributions to this area of research. The heat map indicates that the Americas are the continent with the highest concentration of research on this issue, comprising 52% of the papers. This data indicates a distinct trend in research within the United States, South Africa, and Nigeria, suggesting that these nations are at the forefront in examining libraries as venues for fostering social inclusion. Nonetheless, it is evident that investigations on this research issue are conducted on every continent, signifying a worldwide interest in this field of study.

### 3.2 Perspectives on library-based inclusive communities

According to the review, twenty articles, accounting for 20% of the total of 69 analyzed, focus on inclusive communities within libraries. The authors define an inclusive community as one that ensures equitable access to social, educational, and cultural resources, regardless of age, gender, race, nationality, socioeconomic status, or cultural background. Such communities promote active participation and social integration, particularly for groups vulnerable to social exclusion, including older adults, unemployed individuals, immigrants, and persons with disabilities. ^9– 13^ Similarly, the authors observe that libraries have transformed from merely being repositories of books to becoming accessible venues that foster community development. ^5, 14, 15^

Jaramillo ^16^ illustrates that public libraries serve a significant function in Colombia’s post-war period, as they are regarded as optimal environments for fostering social inclusion, particularly for individuals affected by the Colombian armed conflict. Libraries facilitate peacebuilding in crisis zones and reinforce the social cohesion among adjacent communities. The author perceives the library as a secure environment for debate and reconciliation among individuals affected by the Colombian conflict. Individuals engaged in the armed war are presently stigmatized, and the communities that directly experienced the battle frequently harbor a negative perception of individuals involved due to the losses they sustained. Consequently, libraries facilitate peacebuilding by addressing stigma, fear, and disparities. ^4, 16^

Furthermore, 5 of the 69 publications, or 7.25%, addressed inclusion from a wide range of perspectives, including digital inclusion and bridging the digital gap in disadvantaged communities. ^17– 19^ It was also discovered that researchers addressed topics related to community participation through public library strategies and activities, and how these activities can empower the community and foster community cohesion, as well as showcase the full range of library services beyond simply storing books. ^20– 22^

Kerr and Hopkins ^23^ emphasize that discussions of social inclusion in relation to libraries should be understood in the broadest sense, encompassing all types of communities that have experienced marginalization or exclusion by society at various times. This encompasses individuals with physical and/or cognitive disabilities, those residing in excluded or marginalized regions, the LGBTQ+ community, individuals of diverse races, cultures, and religions, the indigenous population, immigrants, and those facing economic challenges. ^12^ Prihatin et. al. ^5^ propose a perspective that emphasizes the necessity of social inclusion to acknowledge and appreciate the diversity of its members, aiming to create opportunities for skill enhancement and improved life choices, thereby enabling individuals to realize their aspirations. Libraries, whether public or private, serve as essential institutions that provide access to information and knowledge, making them suitable environments for typically marginalized communities to seek improvement and find safety. ^20, 23– 26^

Some articles highlight the need to acknowledge that libraries have evolved beyond mere repositories for books and study materials. It is essential to understand that libraries play a crucial role in promoting social inclusion, as they facilitate social and political change. Libraries consistently strive to enhance access to knowledge and mitigate biases against vulnerable communities. ^4, 16, 27– 30^

### 3.3 Infrastructure and accessibility for inclusive communities inside libraries

Regarding infrastructure and accessibility, ten articles (14.49%) of the total sixty-nine articles examined this subject. Given that various authors within the articles, such as Phukubje and Ngoepe ^31^ and Alcívar et al., ^4^ emphasize the significance of spaces designed in accordance with user requirements, it is deemed essential to address this matter. Infrastructure must be tailored to meet the requirements of library users, particularly those with physical disabilities, such as individuals who use wheelchairs. Libraries are required to have staircases to ensure accessibility, and books as well as other materials must be positioned at a height accessible to all users, including young children. ^4, 31, 32^

Accessibility encompasses not only the means of accessing library facilities but also the availability of activities, service routes, and digital services to all individuals. ^11, 17, 18^ Libraries ought to evaluate their structural design, as many do not provide easily accessible routes for diverse users, which obstructs entry and limits awareness of available services and activities. ^4, 33, 34^ In this context, training for library staff is crucial to ensure optimal service delivery to individuals with disabilities. ^31^ Consequently, the literature identifies these two barriers as the primary obstacles to achieving effective inclusion in libraries. ^30, 33^

Pomputius ^18^ and Lorbeer ^19^ assert that in discussions of infrastructure and accessibility, libraries must establish accessible digital frameworks for individuals with visual impairments. This necessitates the implementation of specialized digital tools to facilitate access to online library services, including Braille books and audiobooks, ensuring equitable access to knowledge for visually impaired individuals. ^22, 24, 35, 36^ Infrastructure and accessibility for those with hearing disabilities must be considered to ensure their full enjoyment of all library services. Consequently, it is imperative for personnel (librarians, managers, directors, etc.) to communicate proficiently with users, providing information and services to the community via sign language videos or assistive technologies. ^3, 27, 32, 35, 36^

Moreover, it is imperative to note that technological accessibility must extend to users in rural areas, where the library may serve as the sole venue for free internet access. Similarly, libraries, in their role of promoting social inclusion, assist in mitigating the digital divide in rural or remote regions, as the absence of reliable and cost-effective broadband exacerbates inequality in this domain, particularly when access to certain library services is increasingly incorporated into the library’s website. ^15, 25, 37– 40^

In the current era, considering the trend observed within the articles—where six articles (8.7%) address this topic and reflect the authors’ perspectives—it is not sufficient to evaluate the infrastructure and accessibility of a location solely based on the perceptions of frequent users who physically utilize the library space and the librarians. It is also essential to incorporate the perceptions of social media users, who, despite not being physically present, can assess accessibility in terms of structural features. Accessibility is now assessed based on public perception among internet users, taking into account the physical environment, connectivity within social networks to engage the community with the library’s structure, and the digital reputation as reflected in user reviews online. These indicators are employed to evaluate the perceived accessibility from the users’ perspective. ^10, 7, 37, 41– 46^

Civallero ^47^ asserts that accessibility and infrastructure encompass not just physical environments for individuals with disabilities but also symbolic spaces and cultural inclusion, which are vital for attaining comprehensive accessibility for all users. This encompasses indigenous groups with unique traditions and symbols that should be integrated into libraries for comprehensive accessibility; likewise, certain religions have practices that must be acknowledged and honored. Consequently, it is imperative that libraries refrain from enforcing cultural norms and instead evolve into venues for cultural exchange and collaboration, accommodating the diverse customs and traditions of library patrons. ^23, 48– 52^ Moreover, accessibility is predicated on variety, multiplicity, inclusion, and equality. Libraries aim to further these principles along with social inclusion, as they are regarded as institutions of social justice. Libraries aim to rectify discrepancies in service access experienced by specific communities, striving to eliminate disparities among these populations. ^25, 42, 50^

### 3.4 Libraries’ community engagement

Twenty-five articles address community engagement, constituting 36.23% of the overall articles. Bangani et al. ^14^ assert that library participation constitutes the initial step toward attaining social inclusion. By dedicating themselves to their community, libraries will consistently endeavor to guarantee that all users feel included and that their services remain pertinent and accessible to all. ^3, 16, 21, 23, 42, 48, 49^

To accomplish this, libraries must utilize co-creation spaces with their communities to ascertain their genuine needs. Blackburn ^53^ indicates in his case study that authentic collaboration between indigenous and aboriginal communities and public libraries in the Torres Strait successfully transcended cultural, social, and structural barriers, thereby enabling co-creation spaces to assist libraries in developing services tailored to the requirements of the island’s indigenous and aboriginal community. Similarly, community engagement fosters the professional growth of librarians by imparting essential insights regarding the community’s needs and interests as users. ^4, 5, 7, 9, 47, 48, 52, 54^

The obligations of each library differ based on its specific setting. Each library possesses its unique setting and challenges, as indicated by the research findings presented in the articles. The community engagement of an academic library contrasts with that of a rural library. An academic library should prioritize specialized content pertinent to its academic discipline, and its services should be designed to assist students with their scholarly inquiries. Rural libraries must encompass a diverse array of content, ranging from children’s resources to materials for senior citizens, while prioritizing access to various information and community engagement initiatives for their patrons. ^7, 14, 20^

For example, the Oakland University William Beaumont School of Medicine Medical Library implemented the “Integrate-Partner-Create” method. This technique enabled a flexible and sustainable distribution of labor and resources. Partnerships with other institutions also helped to ensure that this strategy was successfully implemented. Public health programs, continuing medical education, and community medical literacy are all vital activities. This technique was conceived and developed to be applied by a variety of libraries. ^55^ Another example of the authors’ proposed tactics for demonstrating community support to libraries is what happened in South and Central Appalachian libraries. The libraries established a community garden in their towns, as well as a seed library and agricultural education programs. This technique improved community cohesion and food security, demonstrating the community’s dedication to its development and well-being. ^3, 10, 28, 48, 56^

Community engagement involves numerous things, the most important of which is community education in order for the library to remain committed to its patrons. In this situation, a research by Bangani ^21^ discusses how libraries promote inclusion and eliminate socioeconomic disparities through education. Furthermore, libraries promote and participate in initiatives such as donating books, clothing, and school shoes to local children, delivering digital workshops, and assisting rural schools near the library. This highlights how libraries can be valuable allies in supporting and promoting community and social education in their areas. Libraries demonstrate social responsibility by supporting their communities in various ways. ^9, 16, 20, 27, 55, 57, 58^

Ultimately, it is essential to examine the authors’ perspective on the role of libraries in facilitating transparent and accurate access to information, as eight of the analyzed publications focus on this subject, constituting 11.59% of the total articles assessed. These articles pertain to the political and democratic dimensions that pervade the communal context. Various articles examine these challenges across time and their impact on the community, enabling libraries to serve as catalysts for civic development, fostering citizen engagement and community empowerment. This illustrates libraries’ dedication to their community by offering quality and dependable information, enabling individuals to comprehend the present political scene. They aim to foster an informed and engaged society within the political sphere, enabling residents to comprehend the impact and advantages of governmental actions. ^27, 50^ Moreover, the writers emphasize the importance of libraries in maintaining relevance through their proactive engagement with their communities. Libraries endeavor to integrate adolescents with various community groups to foster social inclusion, demonstrating creativity and resourcefulness in assisting their communities through unconventional techniques, such as haunted homes and pottery painting, despite limited resources. Relocating libraries to unconventional venues exemplifies the dedication and engagement of librarians towards their community. ^21, 27, 48, 59^

### 3.5 Bridges for institutional collaboration and transformation

Thirteen of the examined articles address this subject, constituting 18.84% of the total 69 articles. Sánchez-García and Yubero ^9^ elucidate that for libraries to effectively foster social inclusion and support their users, they require allies to enhance their influence and demonstrate their advancements and strategies to the world. Collaborations with other institutions are essential for libraries to extend their reach to marginalized communities and address complex issues such as inequality, digital exclusion, and education. Increased collaboration facilitates the identification of solutions and strategies to tackle these challenges. Furthermore, libraries are increasingly recognized as hubs of social capital that effectively connect resources and individuals. ^7, 14, 16, 21, 45, 60^

Moreover, numerous studies across all disciplines indicate that libraries must collaborate with multiple institutions, departments, and organizational units, including medical libraries, hospitals, and directly with physicians or health associations, to have influence. This is executed to enhance public health in communities with the assistance of physicians. ^44, 55^ The objective is to enhance awareness of cancer throughout the community, including detection methods and initial procedures to take upon suspicion. Ultimately, it was determined that the early detection rate improved in locations where these collaborative institutional seminars were conducted. This example illustrates that libraries, via institutional partnership, effectuate beneficial transformations in communities, specifically highlighting the improvements in community health resulting from the conducted workshops. It further reinforces the notion that libraries possess a multifaceted role that extends far beyond merely housing books and facilitating access to knowledge for the community. Libraries serve a social function that promotes community development and cultivates inclusive environments that mitigate social disparities. ^14, 21, 23, 44, 56, 58^

McDowell ^61^ contends that in discussions of collaboration, it is insufficient to concentrate solely on organizations; the role of librarians and their approaches to interorganizational collaborations are equally vital. Additionally, four of the articles examined pertain to this subject, accounting for 5.8% of the total articles. Collaboration is an essential competency in the professional environment, particularly for librarians, and it is vital that they develop this skill. It is essential for librarians to develop the ability to collaborate with various organizations and operate in environments outside the library in order to effectively engage with the community. Librarians must extend their efforts beyond the confines of the library and engage in collaborations with a variety of organizations to broaden their community service network and create a meaningful impact. Similarly, it is essential to recognize that libraries possess the full capacity to serve as catalysts for social change and can achieve this role when their librarians are adequately trained and dedicated to fostering sustainable alliances that promote social inclusion. ^23, 27, 34, 40, 47, 60– 62^

Numerous studies have demonstrated that inter-institutional collaboration includes partnerships with community organizations adjacent to libraries. When libraries collaborate with community organizations, they prioritize the genuine needs of the community and strive to identify effective solutions. A study by Mehra et al. ^24^ in the South and Central Appalachian region indicates that community organizations and libraries collaborated to address the technological divide in these regions. The groups and libraries collaborated to establish school literacy initiatives, online learning platforms, and digital tools to engage individuals at risk of exclusion. There is also discussion over the customization of technology to align with community requirements and socioeconomic demands. This continuous collaboration illustrates that libraries cannot operate in isolation from other entities, and without such partnerships, numerous projects and strategies would remain unimplemented. ^53, 63, 64^

## 4. Discussion

In recent years, libraries have evolved from simple repository of books and information into environments that promote learning and social transformation, facilitating growth and inclusiveness. Libraries serve as accessible and impartial environments intended to foster the development of communities that advocate for the inclusion of historically disadvantaged populations. This review of 69 articles published from 2014 to 2024 aimed to ascertain how scientific data pertains to the design of public libraries as environments that foster social inclusion.

The findings reveal that the predominant volume of literature concerning social inclusion in libraries was generated between 2020 and 2024, accounting for 44% of the analyzed publications. The data indicates a growing interest in the topic throughout time, alongside an expansion in geographic locations for data collecting and the advancement of inclusion initiatives. The Americas, especially the United States, exert the most significant impact and produce the highest volume of scholarly publications. Nations like Nigeria and South Africa have demonstrated interest in inclusion and have experienced academic advancement in the generation of articles and research on this subject. Nevertheless, several nations continue to exhibit insufficient advancement in research and publication, thereby lagging in a world that increasingly prioritizes community inclusion.

The countries conducting substantial research on libraries as spaces to foster social inclusion predominantly adopt diverse perspectives, ranging from institutional collaboration to viewing libraries as venues for social justice. All of these approaches emphasize how libraries serve as inclusive and secure environments for communities that have experienced violations or marginalization by society.

Numerous scholars concur that collaboration between libraries and various public or private entities is essential for sustaining the role of libraries as centers of inclusion. This subject is covered in 18.84% of the articles, a notable statistic, primarily because inter-institutional collaboration is essential for the feasibility of numerous strategies and initiatives executed in libraries globally. Libraries cannot operate in isolation from other institutions, as inclusion necessitates the integration of varied resources and individuals who may offer education and opportunity to those who have been excluded and marginalized. This can only be accomplished if libraries and institutions, including schools, governments, medical centers, and local organizations, collaborate and provide assistance to empower and influence individuals who face persistent rejection. ^5, 9, 27, 28, 32, 45, 57, 65^

Authors like Pomputius ^18^ assert that for a location to be inclusive, it must be accessible in all dimensions, encompassing physical, digital, and communicative accessibility for people with mobility, visual, or aural impairments. All these factors must be considered while developing physical and digital environments. Similarly, the technology employed in library environments must undergo certain adaptations to assist those with visual and aural impairments, and it is imperative that librarians receive professional training to address the distinct requirements of persons with disabilities. It is essential to recognize the necessity for culturally accessible environments for individuals of diverse religions and cultures, where they are neither excluded nor stigmatized and can experience a sense of safety. Librarians must be educated to respectfully assist individuals from many cultural and religious backgrounds. ^4, 18, 31– 33, 47, 66^

Research suggests that libraries must occasionally cede authority to communities and collaborate with them to ascertain their most significant needs. Consequently, they can initiate the formulation of strategies and programs that facilitate community growth and enhancement in alignment with its needs and capacities. Education and digital literacy are among the most prevalent and essential strategies for fostering inclusion. These tactics are employed in both urban and rural libraries, as well as in public and private institutions, albeit with distinct variations. Rural libraries, specifically, formulate plans for diverse communities, encompassing indigenous populations, migrants, and individuals with impairments. Collaborating with communities will enable libraries to expand and foster a sense of support and recognition among community members, thereby enhancing the security and trust that communities place in libraries. ^7, 15, 21, 22, 38, 47, 53, 66^

As several authors have noted in their research, for inclusion in libraries to be genuine and practical, librarians must possess expertise in equity, diversity, and inclusion, along with an understanding of inter-institutional collaboration. This is to enable librarians to develop spaces, services, and strategies that encompass all individuals who are regarded as marginalized or excluded. Similarly, training librarians in inter-institutional collaboration enhances the relevance and impact of library activities and strategies within communities and society at large, as it enables the community to become aware of all the services offered by the library and motivates librarians to pursue ongoing improvement. Likewise, inter-institutional collaboration facilitates the acquisition of both financial and human resources, thereby enabling the development and reinforcement of libraries and community strategies. ^3, 56, 61– 65^

To progress, we must recognize that libraries should function as hubs and catalysts for social change. In addition to serving as guardians of literature and knowledge access, they must proactively combat bigotry and exclusion. They must also endeavor to advance inclusion and equity for all individuals. Advancing equity and inclusion in libraries will demonstrate to marginalized groups that these institutions are their supporters and align with their objectives: fostering inclusion to eradicate exclusion and prejudice. Libraries accomplish this via their community efforts and the resultant impact when marginalized communities are ultimately integrated. It similarly examines the actions libraries undertake when society collectively advocates against the discrimination they have endured; in this context, libraries opt to modify their services and policies in an effort to mitigate exclusion and discrimination. ^27, 34, 47, 65, 67, 68^

This review demonstrates that libraries are increasingly dedicated to serving as centers of social inclusion, functioning not only as physical venues for community engagement but also as proactive agents of social transformation, cultural acceptance, and equitable access to education for individuals irrespective of their culture, religion, or socioeconomic status. ^5, 16, 23, 33, 41, 45, 47^ In the examined research, the authors concur that libraries must meticulously devise activities and strategies that foster inclusion in order to effectuate substantial change and impact. This must consider both the physical infrastructure and the spiritual or cultural places accessible to all populations. Libraries must take into account the involvement and perspectives of the communities they want to assist, as their contributions are essential for effecting genuine change. Moreover, libraries should promote inter-institutional collaboration to engage additional groups and formulate enhanced methods. Similarly, to effectuate change, libraries must educate their librarians in diversity and inclusion to ensure they can support all users and deliver optimal services without engaging in discrimination stemming from ignorance. ^10, 14, 17, 48, 49^

This is accomplished through various initiatives undertaken by libraries, including educational and digital literacy programs for all communities, as well as co-creation spaces that foster community engagement. Consequently, libraries evolve from mere repositories of books to inclusive and welcoming environments for communities. ^3, 7, 32, 64, 68– 70^ Nonetheless, libraries continue to encounter obstacles, including the assessment of their strategic impact, the assurance of sustainability, and the necessity for policies that endorse these strategies and initiatives to evolve into venues for social inclusion development. ^4, 45, 71^ The authors concur that libraries must address community needs while also proactively and ethically anticipating those needs, thereby cultivating environments where inclusion, equity, and diversity are regarded as essential societal values. ^9, 48, 64, 65^

## 5. Conclusion

Libraries, originally established as venues for information access, have evolved into catalysts for social transformation, as seen by Prihatin et al. ^5^ This review article examines the influence of libraries in fostering inclusivity. This analysis emphasizes that libraries serve not merely as information access points, but also as educational environments that ensure equitable technological access, foster community dialogue and participation, and support the creation of activities and services for the inclusion of all communities. This has underscored the necessity for enhanced resources and coordination, which would promote chances for social inclusion and community engagement.

Moreover, it is essential to encourage additional study in nations with little or nonexistent studies in this domain, facilitating an evaluation of how libraries have evolved to promote inclusion across diverse contexts. From this viewpoint, it is essential to enhance empirical research that investigates how libraries foster inclusive practices, especially in rural regions and among marginalized or excluded groups, including Indigenous populations, low socioeconomic communities, and individuals with disabilities. Furthermore, it is imperative to consider inclusion in libraries from all perspectives, not solely infrastructure and participation, to offer a comprehensive area of inquiry for scholars and practitioners focused on their advancement.

## Data Availability

Rodriguez, S. P., Vargas, A. D., Rodriguez, D. E., & Niño, C. A. Extended Data: Libraries and Inclusive Communities. *Zenodo.* **2025**. https://doi.org/10.5281/zenodo.17728740.
