# Libraries as Spaces for Inclusive Communities: A Literature Review

**DOI:** 10.12688/f1000research.175122.2

**Published:** 2026-07-01

**Authors:** Salma Paola Rodríguez Poveda, Ana Dolores Vargas Sánchez, Diego Efrén Rodríguez Cárdenas, Cesar Augusto Niño Hernández

**Affiliations:** 1 Universidad de La Sabana, Chia, Cundinamarca, Colombia

**Keywords:** Library, Inclusivity, Community, knowledge, Education

## Abstract

Recognizing and appreciating the diversity of people around the world is a necessary step in achieving equality for all. The opening of equitable chances for society as a whole is one of the ways in which libraries contribute to the reduction of the exclusion gap. Despite their expanding relevance, institutions like libraries demonstrate inconsistent patterns of integration and research. This literature analysis aimed to examine the methods employed by public libraries to foster social inclusion across various populations from 2014 to 2024. A comprehensive systematic literature review was performed, identifying n = 69 interventions that fulfilled the inclusion criteria according to PRISMA methodological principles. The findings indicate a necessity for collaboration between libraries and other organizations to enhance their effectiveness in promoting social inclusion. The findings underscore the necessity of adopting a comprehensive perspective on inclusion in libraries, which encompasses an examination of infrastructure and participation in physical spaces, as well as additional aspects and dimensions, integrating both physical and digital inclusion.

## 1. Introduction

Today, inclusion in the globe is a concept and a social change trend that is widely seen from various viewpoints, since it entails recognising and appreciating human uniqueness and pursuing equal chances for everyone. Libraries, for their part, provide open and free access to information and culture for the entire community, regardless of color, gender, age, social or economic background, or educational level, among other things. Libraries can now assist close the exclusion gap by providing equal access to information.

Ainscow ^1^ states that for thirty years, numerous initiatives have been undertaken to advance inclusion in educational settings, typically according to a philosophy known as education for all. To attain this objective, it is essential to not only enhance schools but also to include the entire community. In this context, public libraries, which engage in non-academic educational activities, serve as an appropriate venue for fostering inclusivity.

Inclusion has been characterized in various ways. Pionke et al. ^2^ state that it involves the act of establishing secure environments or spaces where individuals feel included, appreciated, and respected. In this context, when discussing inclusion, we are referring to an environment that is accessible to all individuals, free from any form of discrimination. Libraries, in particular, serve as environments that foster such opportunities, possessing both the personnel and the space necessary to facilitate inclusive experiences. Furthermore, libraries were established with the intent of serving as repositories of knowledge. Their role is to facilitate information retrieval for individuals and to ensure that these services are accessible to all (Milano, 2013 cited in ^3^).

In the past decade, research has examined how libraries have approached inclusion from several viewpoints, including digital literacy and accessibility for individuals with impairments. The ethical and social function of librarians as facilitators of social inclusion has been examined. ^3^ Due to swift technology advancement, libraries have had to adjust, enabling them to restructure for enhanced inclusivity in all dimensions.

Alcívar et. al. ^4^ contend that the Marrakesh Treaty (2013) facilitates access to books and other physical materials in libraries for individuals who, due to visual or physical disabilities, are unable to utilize them in their original version. The treaty permits the alteration of copyright laws to transcribe materials into Braille or other formats, so enhancing access to information and knowledge for individuals with impairments.

Libraries, as public and cultural institutions, reassert their role as crucial spaces for promoting inclusion and cohesiveness among varied populations as a result of the widespread social trend of inclusion. As a result, it is regarded as an important field of study and analysis. There are still differences between what is written in the literature and what is actually done in libraries, even with the abundance of published studies. Replicating tactics that are adaptable to other contexts is made possible by an understanding of the projects and research conducted in libraries, especially in rural areas. ^5, 6^ However, by examining the tactics that libraries have started to use, it is possible to recognise the invisible obstacles that make inclusion challenging and to see which of their daily routines are exclusionary, even if that is not their intention. ^7, 8^ According to Prihatin et al. ^5^ and Jaramillo, ^9^ rural libraries act as places of sanctuary for vulnerable individuals or communities during times of conflict, making them catalysts for social transformation through inclusive initiatives. Libraries can therefore contribute to the development of a social impact because, in addition to concentrating on the books on their shelves, they engage with their patrons and encourage inclusivity through technology, accessible spaces for everyone, and cooperation with other organisations to bring about change in their communities. ^10^

This article aims to examine the scientific data concerning the role of public libraries as environments that foster social inclusion. This study aims to address the research question: How have public libraries executed strategies and initiatives to foster social inclusion?

Thus, in a second stage, we strive to examine the strategy adopted by theorists and their varied contributions to the subject, seeking to learn from the literature the structured approach to controlling inclusion procedures from libraries. It is envisaged that this review will provide a critical and intelligible synthesis that will contribute to academic and research domains. It is also intended that it would help with future studies, where it will be possible to look at ways to make things better and how libraries help communities.

## 2. Method

The existing literature on the role of libraries as centers for community inclusion worldwide was critically evaluated and synthesised through a systematic literature review, which was the method used to conduct this study. A qualitative approach was employed to synthesise and critically evaluate existing evidence on the specific topic addressed in the literature during the systematic literature review, which also involved the consideration of emergent categories during the data review.

### 2.1 Search criteria

The search covered the years 2014-2024. This decade was chosen for the review because it is when inclusion began to infiltrate communal settings more strongly, shifting from legal or theoretical methods to practical applications relevant to modern society. “But it is no longer enough to adapt to this new digital landscape; it is now essential to make a shift both conceptually and in practice” ^11^ (p. 105). It also looked at how, throughout this period, libraries played a role in community inclusion, becoming what some authors referred to as “social capital partners” ^11^ (p. 103).

The inquiry was performed utilizing the SCOPUS database. This database was chosen due to its status as one of the most comprehensive sources, recognized for the accuracy and dependability of its data. ^12^ The review encompassed articles written in both Spanish and English, while excluding books, book chapters, conference papers, and meta-analyses.

The study concentrated on libraries broadly, and upon reviewing the articles, after excluding those based on language and document type, 200 articles pertinent to the guiding issue were first identified; however, on June 3, 2025, only 198 papers remained without any further exclusions applied.

### 2.2 Source identification

The research was executed in compliance with the PRISMA statement (Preferred Reporting Items for Systematic Reviews and Meta-Analyses). A database search was conducted utilizing the following concatenated keywords: Library AND Inclusive Communities AND Initiative, to encompass all relevant facets of this review issue and enable the incorporation of diverse studies.

The following search equation was entered into the database SCOPUS: (TITLE-ABS-KEY (“library” OR “libraries”) AND TITLE-ABS-KEY (“inclusive communities” OR “social inclusion” OR “diverse communities” OR “access to information” OR “community engagement”) AND TITLE-ABS-KEY (“program*” OR “initiative*” OR “strategy” OR “services” OR “education”)) AND PUBYEAR > 2013 AND PUBYEAR < 2025 AND PUBYEAR > 2013 AND PUBYEAR < 2025 AND (LIMIT-TO (SUBJAREA,“ARTS”) OR LIMIT-TO (SUBJAREA,“DECI”) OR LIMIT-TO (SUBJAREA,“SOCI”) OR LIMIT-TO (SUBJAREA,“PSYC”)) AND (LIMIT-TO (DOCTYPE,“ar”)) AND (LIMIT-TO (EXACTKEYWORD,“Libraries”) OR LIMIT-TO (EXACTKEYWORD,“Library”) OR LIMIT-TO (EXACTKEYWORD,“Academic Libraries”) OR LIMIT-TO (EXACTKEYWORD,“Library Services”) OR LIMIT-TO (EXACTKEYWORD,“Accessibility”) OR LIMIT-TO (EXACTKEYWORD,“Inclusion”) OR LIMIT-TO (EXACTKEYWORD,“Sustainable Development Goals”) OR LIMIT-TO (EXACTKEYWORD,“Community Development”) OR LIMIT-TO (EXACTKEYWORD,“Community-Institutional Relations”) OR LIMIT-TO (EXACTKEYWORD,“Rural Libraries”) OR LIMIT-TO (EXACTKEYWORD,“Libraries, Digital”) OR LIMIT-TO (EXACTKEYWORD,“Library Associations”) OR LIMIT-TO (EXACTKEYWORD,“Equity”) OR LIMIT-TO (EXACTKEYWORD,“Academic Library”)) AND (LIMIT-TO (LANGUAGE,“English”) OR LIMIT-TO (LANGUAGE,“Spanish”).

The recommended inclusion criteria were as follows: accomplished works from the following subject areas: Social Sciences, Arts and Humanities, Decision Sciences, and Psychology, publication dates between 2014 and 2024, and English or Spanish language. Libraries, accessibility, inclusion, community development, equity, rural libraries, and digital libraries were among the keywords employed. The exclusion criteria were as follows: works that were not completed, not directly related to the topics of the systematic literature review, works that only addressed the administrative aspects of libraries rather than their social work, and works that did not provide access to the full text.

### 2.3 Data extraction and analysis

The preliminary search produced 2,120 items. Subsequent to the initial search, exclusion criteria were defined: title, absence of open access, and insufficient relevance to the research during the abstract evaluation, yielding 198 articles.

The authors of this systematic literature review provide a comprehensive and transparent account of the article selection and review process, in accordance with international methodological guidelines. In accordance with the PRISMA 2020 guidelines, ^13^ eligibility and data extraction were conducted independently by distinct reviewers to mitigate bias and enhance the reliability of the selection. The evaluators encountered disagreements during the eligibility and data extraction phases, particularly with respect to the categorisation of the studies. Structured discussions in plenary sessions of the research team were employed to address and resolve these disagreements, resulting in a consensus. This procedure is consistent with the most widely acknowledged methodological best practices, which advocate for the collegial resolution of discrepancies to enhance the reproducibility and validity of systematic reviews.

**Figure 1.**
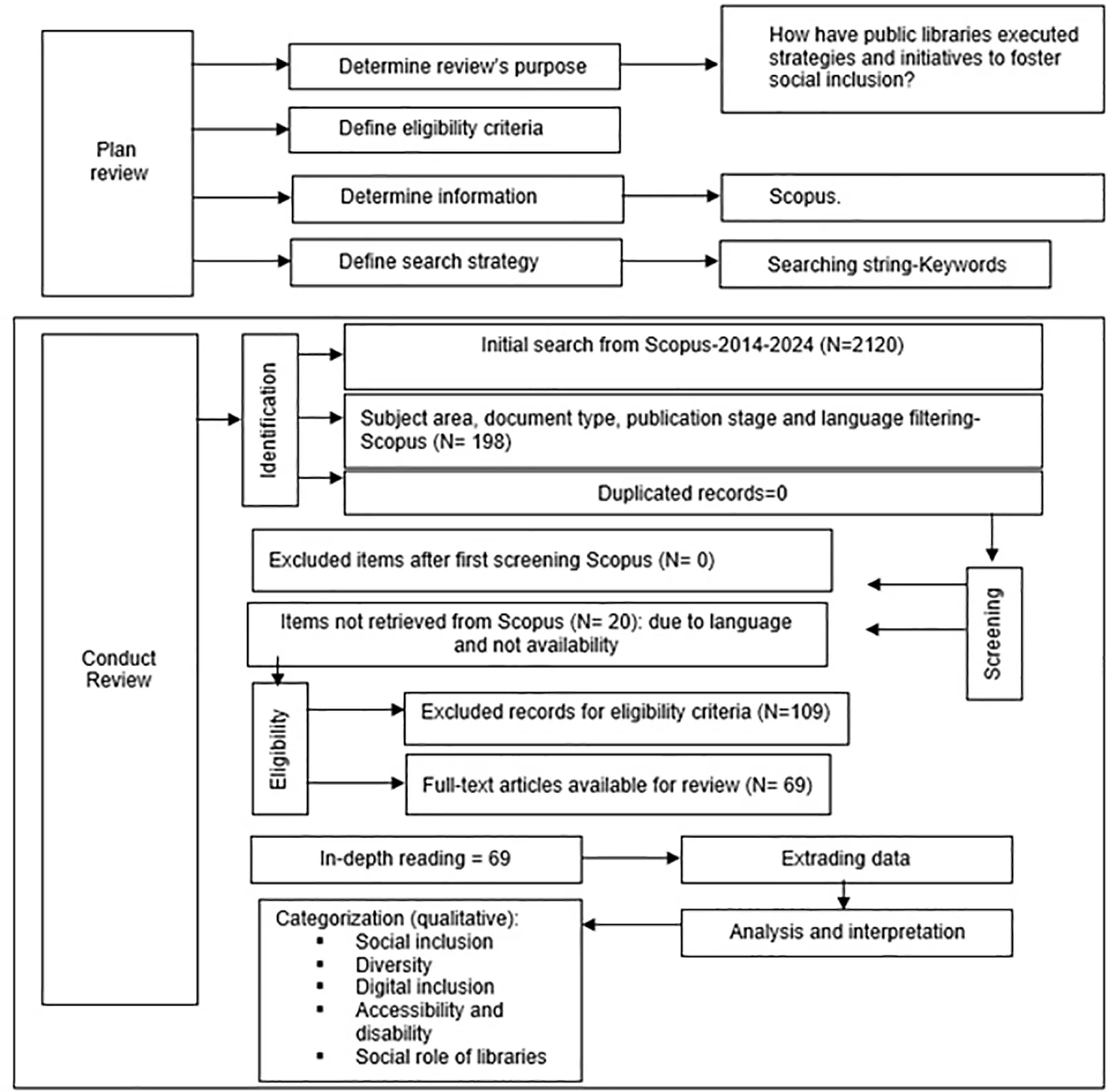
Detailed diagram of information source identification. Source: PRISMA (2020) flow diagram. Prepared by the authors.

Following the elimination of texts that failed to satisfy the inclusion criteria, a total of 69 articles were incorporated into the qualitative analysis. The content analysis included initial reading, coding, delineation of emerging categories, and ultimately, the description and systematization of the data (see Figure 1).

Each article was thoroughly reviewed in its entirety, and recurring patterns were identified through open coding. The preliminary stage of categorization was employed to develop analytical categories and subcategories. This classification facilitated a chronological and thematic analysis to discern patterns, overlaps, and deficiencies. Given the descriptive nature of the review, a formal assessment of risk of bias or methodological quality (e.g., utilizing CASP, AMSTAR, or ROBIS) was not conducted. Therefore, the conclusions derived from the findings should be understood within the scope of the analysis, without relying on categorical generalizations.

The results were systematically categorized thematically and displayed with graphs and heat maps generated using Visme.net and data aggregated in Excel, facilitating the illustration of temporal and geographical trends. All quantitative meta-analyses were eliminated, and aggregate measures of effect and heterogeneity were not calculated, as they beyond the descriptive scope of the study. Publication bias was not addressed; nevertheless, its potential influence is recognized in the limits section. This technique provides a critical qualitative synthesis that amalgamates diverse data, facilitating a greater comprehension of prevalent themes related to inclusion in libraries.

## 3. Results

**Figure 2.**
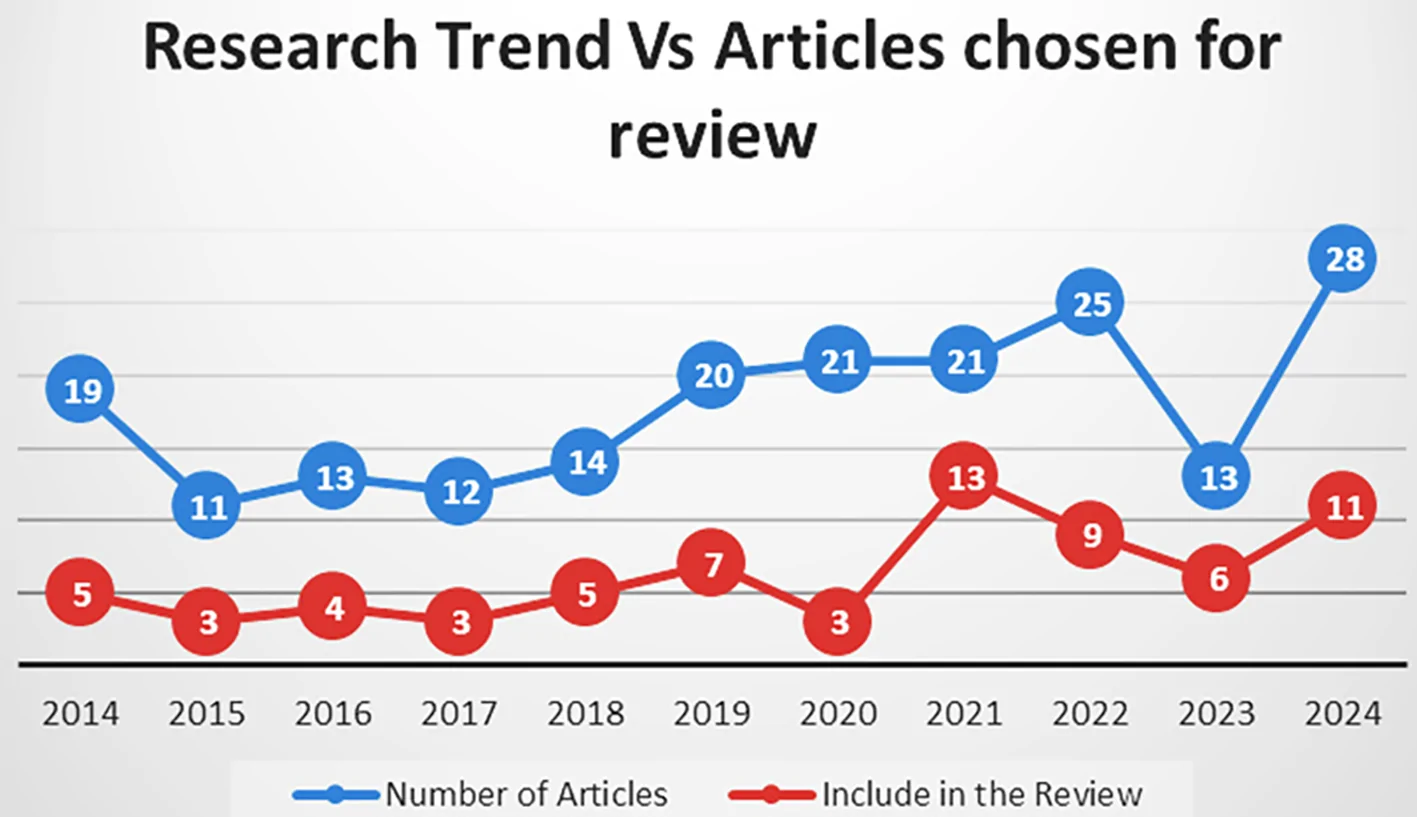
Examination of the research sample. Source: Original composition by the authors.

As evidenced by the time series derived from the documentary corpus, there has been a substantial increase in academic output regarding the enhancement of inclusivity in libraries over the past decade. A reliability rate of 85% was achieved by conducting a statistical calculation to validate the number of studies reviewed. The statistical sample is used to calculate this rate, which is based on the population size (in this instance, the number of articles that were returned from the search) and the margin of error. The reliability level is 85%, with a margin of error of 7% and a population size of 198 articles. Additionally, the inclusion of each article necessitated that at least half of the reviewers concur (refer to Figure 2).

In the year 2014, there was a significant amount of study conducted on libraries as centers for social inclusion. In comparison to 2014, the amount of literature produced on this subject decreased between the years 2015 and 2019, yet it stayed relatively unchanged overall. Between the years 2020 and 2022, there was a spike in the production of literary works, which culminated in a maximum of 25 articles in the year 2022. A significant decrease in the amount of literary work produced took place in the year 2023, with the number of pieces falling from 25 to just 13. Twenty-eight pieces were published in 2024, all of which addressed social inclusion from a variety of angles and examined community participation in libraries. This was the year that literary creation achieved its highest level in the previous ten years.

### 3.1 Geographic distribution

**Figure 3.**
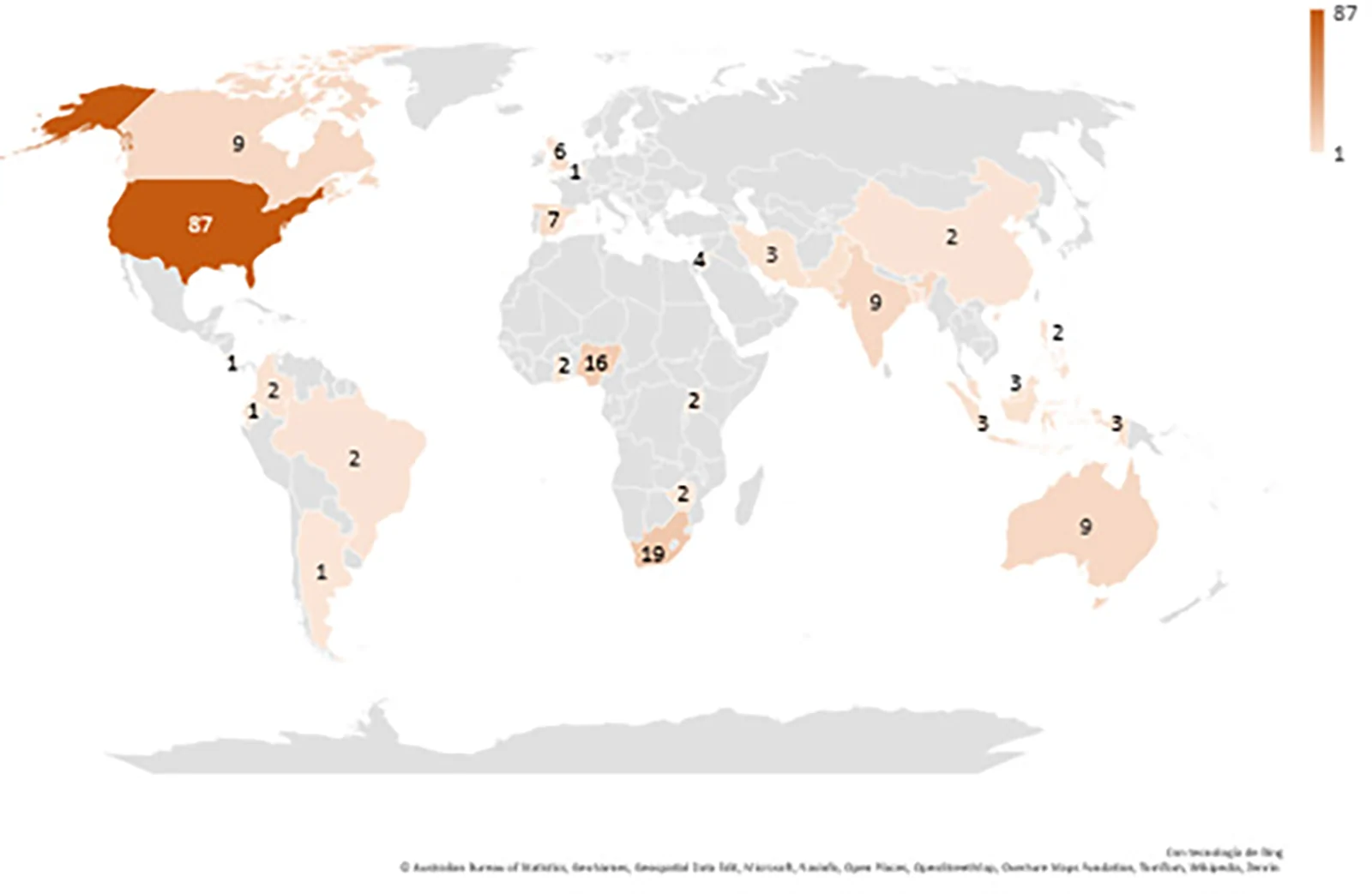
Heatmap of the inquiries. Source: Original composition by the authors.

Figure 3 illustrates a heat map depicting the quantity of papers published by country. The research analyzed were not omitted based on nation but were filtered by language, including only articles in English and Spanish. The research indicates that the scholarly emphasis on libraries’ role in promoting social inclusion is predominantly situated in the United States (87), South Africa (19), Nigeria (16), India (9), Canada (9), and Australia (9), collectively representing 75% of the published papers. Countries including Spain (7), the United Kingdom (6), Jordan (4), Indonesia (3), Iran (3), Malaysia (3), Pakistan (3), China (2), Bangladesh (2), Brazil (2), Colombia (2), Ghana (2), the Philippines (2), Uganda (2), Zimbabwe (2), Argentina (1), Belgium (1), Costa Rica (1), and Ecuador (1) have made substantial contributions to this area of research. The heat map indicates that the Americas are the continent with the highest concentration of research on this issue, comprising 52% of the papers. This data indicates a distinct trend in research within the United States, South Africa, and Nigeria, suggesting that these nations are at the forefront in examining libraries as venues for fostering social inclusion. Nonetheless, it is evident that investigations on this research issue are conducted on every continent, signifying a worldwide interest in this field of study.

### 3.2 Perspectives on library-based inclusive communities

According to the review, twenty articles, accounting for 20% of the total of 69 analyzed, focus on inclusive communities within libraries. The authors define an inclusive community as one that ensures equitable access to social, educational, and cultural resources, regardless of age, gender, race, nationality, socioeconomic status, or cultural background. Such communities promote active participation and social integration, particularly for groups vulnerable to social exclusion, including older adults, unemployed individuals, immigrants, and persons with disabilities. ^10, 14– 17^ Similarly, the authors observe that libraries have transformed from merely being repositories of books to becoming accessible venues that foster community development. ^5, 18, 19^

Jaramillo ^19^ illustrates that public libraries serve a significant function in Colombia’s post-war period, as they are regarded as optimal environments for fostering social inclusion, particularly for individuals affected by the Colombian armed conflict. Libraries facilitate peacebuilding in crisis zones and reinforce the social cohesion among adjacent communities. The author perceives the library as a secure environment for debate and reconciliation among individuals affected by the Colombian conflict. Individuals engaged in the armed war are presently stigmatized, and the communities that directly experienced the battle frequently harbor a negative perception of individuals involved due to the losses they sustained. Consequently, libraries facilitate peacebuilding by addressing stigma, fear, and disparities. ^4, 9^

Furthermore, 5 of the 69 publications, or 7.25%, addressed inclusion from a wide range of perspectives, including digital inclusion and bridging the digital gap in disadvantaged communities. ^20– 22^ It was also discovered that researchers addressed topics related to community participation through public library strategies and activities, and how these activities can empower the community and foster community cohesion, as well as showcase the full range of library services beyond simply storing books. ^23– 25^

Kerr and Hopkins ^7^ emphasize that discussions of social inclusion in relation to libraries should be understood in the broadest sense, encompassing all types of communities that have experienced marginalization or exclusion by society at various times. This encompasses individuals with physical and/or cognitive disabilities, those residing in excluded or marginalized regions, the LGBTQ+ community, individuals of diverse races, cultures, and religions, the indigenous population, immigrants, and those facing economic challenges. ^16^ Prihatin et al. ^5^ propose a perspective that emphasizes the necessity of social inclusion to acknowledge and appreciate the diversity of its members, aiming to create opportunities for skill enhancement and improved life choices, thereby enabling individuals to realize their aspirations. Libraries, whether public or private, serve as essential institutions that provide access to information and knowledge, making them suitable environments for typically marginalized communities to seek improvement and find safety. ^7, 23, 26– 28^

Some articles highlight the need to acknowledge that libraries have evolved beyond mere repositories for books and study materials. It is essential to understand that libraries play a crucial role in promoting social inclusion, as they facilitate social and political change. Libraries consistently strive to enhance access to knowledge and mitigate biases against vulnerable communities. ^4, 9, 29– 32^

### 3.3 Infrastructure and accessibility for inclusive communities inside libraries

Regarding infrastructure and accessibility, ten articles (14.49%) of the total sixty-nine articles examined this subject. Given that various authors within the articles, such as Phukubje and Ngoepe ^33^ and Alcívar et al., ^4^ emphasize the significance of spaces designed in accordance with user requirements, it is deemed essential to address this matter. Infrastructure must be tailored to meet the requirements of library users, particularly those with physical disabilities, such as individuals who use wheelchairs. Libraries are required to have staircases to ensure accessibility, and books as well as other materials must be positioned at a height accessible to all users, including young children. ^4, 8, 33^

Accessibility encompasses not only the means of accessing library facilities but also the availability of activities, service routes, and digital services to all individuals. ^15, 20, 21^ Libraries ought to evaluate their structural design, as many do not provide easily accessible routes for diverse users, which obstructs entry and limits awareness of available services and activities. ^4, 34, 35^ In this context, training for library staff is crucial to ensure optimal service delivery to individuals with disabilities. ^33^ Consequently, the literature identifies these two barriers as the primary obstacles to achieving effective inclusion in libraries. ^32, 34^

Pomputius ^21^ and Lorbeer ^22^ assert that in discussions of infrastructure and accessibility, libraries must establish accessible digital frameworks for individuals with visual impairments. This necessitates the implementation of specialized digital tools to facilitate access to online library services, including Braille books and audiobooks, ensuring equitable access to knowledge for visually impaired individuals. ^25, 26, 36, 37^ Infrastructure and accessibility for those with hearing disabilities must be considered to ensure their full enjoyment of all library services. Consequently, it is imperative for personnel (librarians, managers, directors, etc.) to communicate proficiently with users, providing information and services to the community via sign language videos or assistive technologies. ^3, 8, 29, 36, 37^

Moreover, it is imperative to note that technological accessibility must extend to users in rural areas, where the library may serve as the sole venue for free internet access. Similarly, libraries, in their role of promoting social inclusion, assist in mitigating the digital divide in rural or remote regions, as the absence of reliable and cost-effective broadband exacerbates inequality in this domain, particularly when access to certain library services is increasingly incorporated into the library’s website. ^19, 27, 38– 41^

In the current era, considering the trend observed within the articles—where six articles (8.7%) address this topic and reflect the authors’ perspectives—it is not sufficient to evaluate the infrastructure and accessibility of a location solely based on the perceptions of frequent users who physically utilize the library space and the librarians. It is also essential to incorporate the perceptions of social media users, who, despite not being physically present, can assess accessibility in terms of structural features. Accessibility is now assessed based on public perception among internet users, taking into account the physical environment, connectivity within social networks to engage the community with the library’s structure, and the digital reputation as reflected in user reviews online. These indicators are employed to evaluate the perceived accessibility from the users’ perspective. ^7, 11, 14, 38, 42– 47^

Civallero ^48^ asserts that accessibility and infrastructure encompass not just physical environments for individuals with disabilities but also symbolic spaces and cultural inclusion, which are vital for attaining comprehensive accessibility for all users. This encompasses indigenous groups with unique traditions and symbols that should be integrated into libraries for comprehensive accessibility; likewise, certain religions have practices that must be acknowledged and honored. Consequently, it is imperative that libraries refrain from enforcing cultural norms and instead evolve into venues for cultural exchange and collaboration, accommodating the diverse customs and traditions of library patrons. ^7, 49– 52^ Moreover, accessibility is predicated on variety, multiplicity, inclusion, and equality. Libraries aim to further these principles along with social inclusion, as they are regarded as institutions of social justice. Libraries aim to rectify discrepancies in service access experienced by specific communities, striving to eliminate disparities among these populations. ^27, 43, 50^

### 3.4 Libraries’ community engagement

Twenty-five articles address community engagement, constituting 36.23% of the overall articles. Bangani et al. ^18^ assert that library participation constitutes the initial step toward attaining social inclusion. By dedicating themselves to their community, libraries will consistently endeavor to guarantee that all users feel included and that their services remain pertinent and accessible to all. ^3, 6, 7, 9, 24, 43, 49^

To accomplish this, libraries must utilize co-creation spaces with their communities to ascertain their genuine needs. Blackburn ^53^ indicates in his case study that authentic collaboration between indigenous and aboriginal communities and public libraries in the Torres Strait successfully transcended cultural, social, and structural barriers, thereby enabling co-creation spaces to assist libraries in developing services tailored to the requirements of the island’s indigenous and aboriginal community. Similarly, community engagement fosters the professional growth of librarians by imparting essential insights regarding the community’s needs and interests as users. ^4, 5, 10, 11, 48, 49, 52, 54^

The obligations of each library differ based on its specific setting. Each library possesses its unique setting and challenges, as indicated by the research findings presented in the articles. The community engagement of an academic library contrasts with that of a rural library. An academic library should prioritize specialized content pertinent to its academic discipline, and its services should be designed to assist students with their scholarly inquiries. Rural libraries must encompass a diverse array of content, ranging from children’s resources to materials for senior citizens, while prioritizing access to various information and community engagement initiatives for their patrons. ^11, 18, 23^

For example, the Oakland University William Beaumont School of Medicine Medical Library implemented the “Integrate-Partner-Create” method. This technique enabled a flexible and sustainable distribution of labor and resources. Partnerships with other institutions also helped to ensure that this strategy was successfully implemented. Public health programs, continuing medical education, and community medical literacy are all vital activities. This technique was conceived and developed to be applied by a variety of libraries. ^55^ Another example of the authors’ proposed tactics for demonstrating community support to libraries is what happened in South and Central Appalachian libraries. The libraries established a community garden in their towns, as well as a seed library and agricultural education programs. This technique improved community cohesion and food security, demonstrating the community’s dedication to its development and well-being. ^3, 14, 30, 49, 56^

Community engagement involves numerous things, the most important of which is community education in order for the library to remain committed to its patrons. In this situation, a research by Bangani ^24^ discusses how libraries promote inclusion and eliminate socioeconomic disparities through education. Furthermore, libraries promote and participate in initiatives such as donating books, clothing, and school shoes to local children, delivering digital workshops, and assisting rural schools near the library. This highlights how libraries can be valuable allies in supporting and promoting community and social education in their areas. Libraries demonstrate social responsibility by supporting their communities in various ways. ^9, 10, 23, 29, 55, 57, 58^

Ultimately, it is essential to examine the authors’ perspective on the role of libraries in facilitating transparent and accurate access to information, as eight of the analyzed publications focus on this subject, constituting 11.59% of the total articles assessed. These articles pertain to the political and democratic dimensions that pervade the communal context. Various articles examine these challenges across time and their impact on the community, enabling libraries to serve as catalysts for civic development, fostering citizen engagement and community empowerment. This illustrates libraries’ dedication to their community by offering quality and dependable information, enabling individuals to comprehend the present political scene. They aim to foster an informed and engaged society within the political sphere, enabling residents to comprehend the impact and advantages of governmental actions. ^29, 50^ Moreover, the writers emphasize the importance of libraries in maintaining relevance through their proactive engagement with their communities. Libraries endeavor to integrate adolescents with various community groups to foster social inclusion, demonstrating creativity and resourcefulness in assisting their communities through unconventional techniques, such as haunted homes and pottery painting, despite limited resources. Relocating libraries to unconventional venues exemplifies the dedication and engagement of librarians towards their community. ^24, 29, 49, 59^

### 3.5 Bridges for institutional collaboration and transformation

Thirteen of the examined articles address this subject, constituting 18.84% of the total 69 articles. Sánchez-García and Yubero ^10^ elucidate that for libraries to effectively foster social inclusion and support their users, they require allies to enhance their influence and demonstrate their advancements and strategies to the world. Collaborations with other institutions are essential for libraries to extend their reach to marginalized communities and address complex issues such as inequality, digital exclusion, and education. Increased collaboration facilitates the identification of solutions and strategies to tackle these challenges. Furthermore, libraries are increasingly recognized as hubs of social capital that effectively connect resources and individuals. ^9, 11, 14, 24, 46, 60^

Moreover, numerous studies across all disciplines indicate that libraries must collaborate with multiple institutions, departments, and organizational units, including medical libraries, hospitals, and directly with physicians or health associations, to have influence. This is executed to enhance public health in communities with the assistance of physicians. ^45, 55^ The objective is to enhance awareness of cancer throughout the community, including detection methods and initial procedures to take upon suspicion. Ultimately, it was determined that the early detection rate improved in locations where these collaborative institutional seminars were conducted. This example illustrates that libraries, via institutional partnership, effectuate beneficial transformations in communities, specifically highlighting the improvements in community health resulting from the conducted workshops. It further reinforces the notion that libraries possess a multifaceted role that extends far beyond merely housing books and facilitating access to knowledge for the community. Libraries serve a social function that promotes community development and cultivates inclusive environments that mitigate social disparities. ^7, 18, 24, 45, 56, 58^

McDowell ^61^ contends that in discussions of collaboration, it is insufficient to concentrate solely on organizations; the role of librarians and their approaches to interorganizational collaborations are equally vital. Additionally, four of the articles examined pertain to this subject, accounting for 5.8% of the total articles. Collaboration is an essential competency in the professional environment, particularly for librarians, and it is vital that they develop this skill. It is essential for librarians to develop the ability to collaborate with various organizations and operate in environments outside the library in order to effectively engage with the community. Librarians must extend their efforts beyond the confines of the library and engage in collaborations with a variety of organizations to broaden their community service network and create a meaningful impact. Similarly, it is essential to recognize that libraries possess the full capacity to serve as catalysts for social change and can achieve this role when their librarians are adequately trained and dedicated to fostering sustainable alliances that promote social inclusion. ^27, 29, 35, 41, 48, 60– 62^

Numerous studies have demonstrated that inter-institutional collaboration includes partnerships with community organizations adjacent to libraries. When libraries collaborate with community organizations, they prioritize the genuine needs of the community and strive to identify effective solutions. A study by Mehra et al. ^26^ in the South and Central Appalachian region indicates that community organizations and libraries collaborated to address the technological divide in these regions. The groups and libraries collaborated to establish school literacy initiatives, online learning platforms, and digital tools to engage individuals at risk of exclusion. There is also discussion over the customization of technology to align with community requirements and socioeconomic demands. This continuous collaboration illustrates that libraries cannot operate in isolation from other entities, and without such partnerships, numerous projects and strategies would remain unimplemented. ^53, 63, 64^

## 4. Discussion

In recent years, libraries have evolved from simple repository of books and information into environments that promote learning and social transformation, facilitating growth and inclusiveness. Libraries serve as accessible and impartial environments intended to foster the development of communities that advocate for the inclusion of historically disadvantaged populations. This review of 69 articles published from 2014 to 2024 aimed to ascertain how scientific data pertains to the design of public libraries as environments that foster social inclusion.

The findings reveal that the predominant volume of literature concerning social inclusion in libraries was generated between 2020 and 2024, accounting for 44% of the analyzed publications. The data indicates a growing interest in the topic throughout time, alongside an expansion in geographic locations for data collecting and the advancement of inclusion initiatives. The Americas, especially the United States, exert the most significant impact and produce the highest volume of scholarly publications. Nations like Nigeria and South Africa have demonstrated interest in inclusion and have experienced academic advancement in the generation of articles and research on this subject. Nevertheless, several nations continue to exhibit insufficient advancement in research and publication, thereby lagging in a world that increasingly prioritizes community inclusion.

The countries conducting substantial research on libraries as spaces to foster social inclusion predominantly adopt diverse perspectives, ranging from institutional collaboration to viewing libraries as venues for social justice. All of these approaches emphasize how libraries serve as inclusive and secure environments for communities that have experienced violations or marginalization by society.

The function of libraries as centers of inclusion is contingent upon the collaboration between libraries and a variety of public or private entities, as numerous academics have agreed. This topic is the subject of 18.84% of the articles, which is a remarkable statistic. This is due to the fact that inter-institutional collaboration is essential for the success of a variety of strategies and initiatives that are implemented in libraries worldwide. Libraries cannot function in isolation from other institutions, as inclusion necessitates the integration of a variety of resources and individuals who can provide education and opportunities to those who have been consistently excluded and marginalised by the system, as per the reviewed literature. ^8, 10, 46^ Lara ^29^ argues, in agreement with other authors, that this can only be accomplished through the collaboration and provision of support by libraries and institutions, such as schools, government, medical centers, and local organisations, to empower and influence individuals who encounter persistent rejection. ^5, 29, 30, 55, 65^

According to authors like Pomputius, ^21^ a space must be inclusive in all its dimensions, including physical, digital, and communicative accessibility for individuals with visual, auditory, or mobility impairments. When creating physical and digital environments, it is imperative to take into account all of these factors, as accessibility necessitates the removal of obstacles to enable all individuals to engage in society. ^8, 34^ In the same vein, libraries must modify their technological infrastructure to accommodate individuals with visual and auditory impairments. In the same vein, it is imperative that librarians undergo professional training to address the unique requirements of individuals with disabilities, thereby enabling the implementation of strategies that ensure the right to information for all. ^4, 33^ It is imperative to acknowledge the necessity of culturally accessible environments that are inclusive of individuals of a variety of religions and cultures, where they are not marginalised or stigmatised and can experience a sense of security. ^66^ Librarians must be trained to serve individuals from a variety of cultural and religious contexts with respect, thereby promoting the library as a place for dialogue and encounter that fosters social inclusion. ^48^

Research indicates that libraries should occasionally relinquish authority to communities and work in conjunction with them to identify their most urgent requirements. This is consistent with the concept of community empowerment, which involves the involvement of the public in significant decisions that will have a significant impact on the entire community. ^39, 53^ In this manner, they can begin the co-creation of strategies and programs that promote community growth and development, while prioritising the voices of the community, in accordance with their needs and capabilities. ^66^ Digital literacy and education are among the most prevalent and indispensable strategies for promoting inclusion. The technological disparity between rural and urban environments is mitigated by their action as vehicles. In the same vein, digital inclusion is a critical component of the success of both individuals and communities, and it is imperative that libraries encourage digital literacy. ^11, 19, 24^ Although there are some variations, these strategies are implemented in both public and private institutions, as well as urban and rural libraries. In particular, rural libraries create strategies for diverse communities, such as Indigenous populations, migrants, and individuals with disabilities. ^25, 39, 48^ Libraries are able to expand and cultivate a sense of support and recognition among their members through collaboration with communities, which in turn reinforces the security and trust that communities have in them. ^11, 39, 53^

In order for inclusion in libraries to be both practical and authentic, librarians must possess a comprehensive understanding of inter-institutional collaboration and possess expertise in equity, diversity, and inclusion, as numerous authors have noted in their research. They must transcend passive neutrality and adopt a more proactive approach to the documentation, reporting, and resolution of discrimination in libraries. ^56, 64, 65^ This enables them to create strategies, services, and spaces that are accessible to all individuals who are marginalised or excluded. ^64^ Additionally, the relevance and impact of library activities and strategies in communities and society as a whole are enhanced by the training of librarians in inter-institutional collaboration. This enables librarians to become ambassadors of change by adapting to the social and technological changes that are currently prevalent in society. It enables the community to become informed about the library's full range of services and encourages librarians to pursue ongoing enhancements. Similarly, librarians from various institutions can identify common objectives with the community. ^56, 61– 63^ In the same way, inter-institutional collaboration enables the acquisition of financial and human resources, which in turn enables the development and reinforcement of community strategies and libraries. Similarly, the library is unable to sustain socio-cultural initiatives that require the assistance of partnerships with schools, NGOs, and local leaders. ^3, 61^

In order to advance, it is imperative that we acknowledge that libraries should serve as catalysts and centers for social change, incorporating sociopolitical components to educate and reform the community. ^29^ They must actively counteract intolerance and exclusion, in addition to safeguarding literature and access to knowledge, by disseminating accurate information that combats intolerance, thereby adopting a more dynamic role in defending equity. ^35, 65^ Similarly, they must endeavour to ensure that all individuals have access to information as a public benefit, which contributes to the reduction of inequality, and to promote inclusion and equity. ^29^ The promotion of equity and inclusion in libraries serves as a testament to the institutions’ commitment to marginalised communities and their shared objectives: the elimination of exclusion and prejudice through the promotion of inclusion. This will establish environments in which all voices are acknowledged and appreciated within the library and the community. ^67^ This is accomplished by libraries through their community initiatives and the subsequent impact when marginalised communities are integrated through forums where they discuss their problems and concerns. ^35, 48^ The actions performed by libraries when society mobilises collectively against discrimination are also analysed. Libraries opt to modify their policies and services in order to reduce discrimination and exclusion in this context. In the same vein, libraries must elect to collaborate with government entities in order to adhere to national integration ^35, 68^

This review demonstrates that libraries are increasingly dedicated to serving as centers of social inclusion, functioning not only as physical venues for community engagement but also as proactive agents of social transformation, cultural acceptance, and equitable access to education for individuals irrespective of their culture, religion, or socioeconomic status. ^5, 7, 9, 34, 42, 46, 48^ In the examined research, the authors concur that libraries must meticulously devise activities and strategies that foster inclusion in order to effectuate substantial change and impact. This must consider both the physical infrastructure and the spiritual or cultural places accessible to all populations. Libraries must take into account the involvement and perspectives of the communities they want to assist, as their contributions are essential for effecting genuine change. Moreover, libraries should promote inter-institutional collaboration to engage additional groups and formulate enhanced methods. Similarly, to effectuate change, libraries must educate their librarians in diversity and inclusion to ensure they can support all users and deliver optimal services without engaging in discrimination stemming from ignorance. ^6, 14, 18, 20, 49^

This is accomplished through various initiatives undertaken by libraries, including educational and digital literacy programs for all communities, as well as co-creation spaces that foster community engagement. Consequently, libraries evolve from mere repositories of books to inclusive and welcoming environments for communities. ^3, 8, 11, 64, 67, 69, 70^ Nonetheless, libraries continue to encounter obstacles, including the assessment of their strategic impact, the assurance of sustainability, and the necessity for policies that endorse these strategies and initiatives to evolve into venues for social inclusion development. ^4, 46, 71^ The authors concur that libraries must address community needs while also proactively and ethically anticipating those needs, thereby cultivating environments where inclusion, equity, and diversity are regarded as essential societal values. ^10, 49, 64, 65^

## 5. Conclusion

Libraries, originally established as venues for information access, have evolved into catalysts for social transformation, as seen by Prihatin et al. ^5^ This review article examines the influence of libraries in fostering inclusivity. This analysis emphasizes that libraries serve not merely as information access points, but also as educational environments that ensure equitable technological access, foster community dialogue and participation, and support the creation of activities and services for the inclusion of all communities. This has underscored the necessity for enhanced resources and coordination, which would promote chances for social inclusion and community engagement.

Moreover, it is essential to encourage additional study in nations with little or nonexistent studies in this domain, facilitating an evaluation of how libraries have evolved to promote inclusion across diverse contexts. From this viewpoint, it is essential to enhance empirical research that investigates how libraries foster inclusive practices, especially in rural regions and among marginalized or excluded groups, including Indigenous populations, low socioeconomic communities, and individuals with disabilities. Furthermore, it is imperative to consider inclusion in libraries from all perspectives, not solely infrastructure and participation, to offer a comprehensive area of inquiry for scholars and practitioners focused on their advancement.

## Data Availability

Rodriguez, S. P., Vargas, A. D., Rodriguez, D. E., & Niño, C. A. Extended Data: Libraries and Inclusive Communities. *Zenodo.* **2025**. https://doi.org/10.5281/zenodo.17728740.
