# Supplementary material for: Libraries as Spaces for Inclusive Communities: A Literature Review
Source: F1000Res. 2026 Jul 1;15:43. Originally published 2026 Jan 9. [Version 2] doi: 10.12688/f1000research.175122.2 (PMC13554730; doi:10.12688/f1000research.175122.2)
Supplement: Peer review history

# Reviewer report: Reviewer response for version 2

**DOI:** 10.5256/f1000research.204713.r499682

**Published:** 2026-08-14

**Authors:** Christine Mady

**Affiliations:** 1 Aalto University, Espoo, Finland

This literature review on public libraries as spaces for inclusive communities is a welcome contribution to current research on their contemporary roles. Here are some comments to help develop or clarify some aspects of the manuscript.

 In the abstract, the authors need to make the clear distinction between equality and equity, the two terms are not interchangeable. Given that we are nearing the end of 2026, would it be possible for the authors to include also articles from 2025, noting that new research on public libraries has emerged during 2025 and their shifting societal role, which support but also enhance the findings indicated from the review of 2014 to 2024. Alternatively, the authors could at least indicate this in their conclusion, citing some key references for further exploration along the same theme. After reading the full manuscript, and since the number of articles addressing public libraries and inclusion increased after 2020, it is more evident that at least literature from 2025 should be included in this review, perhaps then rescoping to 2015-2025.

 The keywords might consider culture, since the article is about inclusion and diversity

**Introduction:** though public libraries are inclusive, most require a home address to be able to borrow from its resources. This should be acknowledged when talking about them being all inclusive facilities. Moreover, libraries have moved beyond being depositories of collections, and this body of literature needs to be accounted for in an article that is a literature review on public libraries and inclusion. Key references on libraries within the 2014-2024 are not indicated in the list of references.

 The claim that public libraries promote inclusion and ‘cohesiveness’ needs to be further supported with references.

 It is also important to define the context within which public libraries are studied whether urban, rural, or whether central and peripheral within the urban context, as this could reveal different aspects related to their strategies for inclusion and capacities to sustain inclusion.

**Method:** the methodology is clearly explained, with some notes to be further considered. The justification for choosing the year range is not convincing, inclusion in public facilities and public space predates 2014. If the authors are specifically referring to literature on inclusion in libraries, this needs to be clearly stated and exemplified with evidence. The choice of Scopus and exclusion of books and book chapters needs to be explained, its causes and consequences.

 Under the selection criteria, it is interesting to explain why geography was excluded, or in fact, why any discipline dealing with urban issues was not considered.

**Results:** in reading the variation in publications regarding public libraries and inclusion, it would be relevant to mark any differences in relation to the COVID-19 pandemic and lockdowns that happened in different cities globally, and the possibility of this disruption affecting libraries’ roles.

 The geographic distribution corelates with the publication languages, something the authors could also reflect upon, and examine also in relation to number of researchers in the related fields, if such information is publicly available. Literature from Europe on the theme exists within the indicated period 2014-2024 but is not included in the references.

 The term ‘inclusive community’ would require further explanation in the introduction, or justification indicating that it is used as a term, based on its recurrence in the reviewed articles. This was confusing to read under the results, whereas by reading the introduction, the implicit assumption was that the main theme is on public libraries and inclusion.

 Accessibility in libraries is not limited to physical access, it is also digital access, and library cards that could prevent some individuals from being included. This requires further discussion, and if articles on this aspect were not included, further review is required.

 When referring to users in remote areas (rural being one example), are mobile libraries included in the review?

 The reference to symbolic and cultural inclusion would benefit from further elaboration. This might be one important contribution of this literature review, if sufficiently elaborated.

 In the section on community engagement, the manuscript is already discussing libraries’ roles beyond depositories, though key references on this matter are missing. Community engagement in the governance of libraries could be another important contribution of this manuscript, which requires further elaboration.

 Transparency and accuracy of information shared as one of the library’s roles is a third important contribution, especially in this period of mis- and disinformation globally.

 Collaborations of libraries and other institutions include cultural and educational ones (youth centres, lifelong learning, for example), require further examination and elaboration. This discussion cannot be discerned from exploring libraries’ governance, with their legal, regulatory and financial aspects.

**Discussion:** to further enhance the clarity of the discussion, it would help to include key hypotheses or research questions in the introduction section, along with an indication of the research scope. While ‘inclusion’ is one theme, sub-themes related to the role and nature of the public library could be outlined. Currently, the discussion is more an iteration of the results rather than broader reflections on what public libraries in different capacities (central, peripheral, urban or rural) can offer to enable and sustain inclusion. The term community inclusion could more critically be addressed, along with the other points raised in the comments.

**Conclusion: ** The conclusion requires rewriting. Rather than reiterating what the article did, it could develop what is introduced as directions for further research, yet after indicating the limitations of the current one. In case the authors decide not to change the time interval to 2015-2025, then it is important to include references from 2025 that were excluded yet address the theme of inclusion with novel and pertinent research contributions.

**Formatting:** Revising the manuscript for language editing is needed. The language used in the discussion is quite imperative, and perhaps should be reconsidered, as all decisions on libraries are relational, circumstantial and contextual., rather than absolute and universal.

Are the rationale for, and objectives of, the Systematic Review clearly stated?

Yes

Is the statistical analysis and its interpretation appropriate?

Partly

If this is a Living Systematic Review, is the ‘living’ method appropriate and is the search schedule clearly defined and justified? (‘Living Systematic Review’ or a variation of this term should be included in the title.)

Not applicable

Are sufficient details of the methods and analysis provided to allow replication by others?

Yes

Are the conclusions drawn adequately supported by the results presented in the review?

Partly

Reviewer Expertise:

public spaces, public libraries, urban design, urban planning, urban studies

I confirm that I have read this submission and believe that I have an appropriate level of expertise to confirm that it is of an acceptable scientific standard, however I have significant reservations, as outlined above.

# Reviewer report: Reviewer response for version 2

**DOI:** 10.5256/f1000research.204713.r499675

**Published:** 2026-07-30

**Authors:** Oluwayemi IbukunOluwa Odularu

**Affiliations:** 1 Walter Sisulu University, Mthatha Campus, South Africa

The review stated that 69 studies were included in the final synthesis; however, 71 references appear to relate to the included literature. This discrepancy requires clarification. The number of included studies should align with the evidence synthesized, or the authors should clearly distinguish between included studies and additional background references to ensure methodological transparency.

Are the rationale for, and objectives of, the Systematic Review clearly stated?

Yes

Is the statistical analysis and its interpretation appropriate?

Yes

If this is a Living Systematic Review, is the ‘living’ method appropriate and is the search schedule clearly defined and justified? (‘Living Systematic Review’ or a variation of this term should be included in the title.)

Yes

Are sufficient details of the methods and analysis provided to allow replication by others?

Yes

Are the conclusions drawn adequately supported by the results presented in the review?

Yes

Reviewer Expertise:

Education Curriculum; Library and Information Science; Information and Communication Technology (ICT); Artificial Intelligence (AI) in Education and Library Services; Information Systems; Information Management; Knowledge Management and Sharing; Information Literacy; Electronic Resources; Sustainable Development Goals (SDGs) in Education; Socio-Environmental Impact Assessment; LIS Education; Multidisciplinary Research; Information Literacy and Criminal Justice.

I confirm that I have read this submission and believe that I have an appropriate level of expertise to confirm that it is of an acceptable scientific standard, however I have significant reservations, as outlined above.

# Reviewer report: Reviewer response for version 2

**DOI:** 10.5256/f1000research.204713.r497885

**Published:** 2026-07-20

**Authors:** Weerayut Weerayut Seekhunlio

**Affiliations:** 1 Mahasarakham University, Mahasarakham, Thailand

The authors have addressed the principal concerns raised in the previous review and have improved the transparency of the review process, including the description of screening, eligibility criteria, reviewer responsibilities, data extraction, and the resolution of disagreements. The Discussion and Conclusion have also been revised to provide a more balanced interpretation of the findings and to acknowledge the methodological limitations. The revised manuscript is scientifically sound and suitable for publication. I have no further comments.

Are the rationale for, and objectives of, the Systematic Review clearly stated?

Partly

Is the statistical analysis and its interpretation appropriate?

Partly

If this is a Living Systematic Review, is the ‘living’ method appropriate and is the search schedule clearly defined and justified? (‘Living Systematic Review’ or a variation of this term should be included in the title.)

Not applicable

Are sufficient details of the methods and analysis provided to allow replication by others?

Partly

Are the conclusions drawn adequately supported by the results presented in the review?

Partly

Reviewer Expertise:

Ethnomusicology; Music Education; Cultural Heritage Studies; Qualitative Research Methodology; Arts and Humanities Research

I confirm that I have read this submission and believe that I have an appropriate level of expertise to confirm that it is of an acceptable scientific standard.

# Reviewer report: Reviewer response for version 1

**DOI:** 10.5256/f1000research.193081.r471883

**Published:** 2026-04-11

**Authors:** Weerayut Weerayut Seekhunlio

**Affiliations:** 1 Mahasarakham University, Mahasarakham, Thailand

**Points that must be addressed to make the article scientifically sound**

 First, the authors should clarify the review type. If this is intended to be a systematic review, the title, abstract, and methods should consistently describe it as such, and the methodological standards expected of a systematic review should be met. If the authors prefer to retain the study as a literature review, then claims of PRISMA-guided systematic rigor should be moderated accordingly. Second, the methods section must be strengthened substantially. The authors should explain the full screening process in detail, specify whether more than one reviewer screened records and extracted data, describe how disagreements were resolved, and define the exact inclusion and exclusion criteria in reproducible terms. Third, the exclusion of non-open-access papers should be justified very carefully or removed, because this decision can bias the sample. Fourth, a formal appraisal of methodological quality or risk of bias should be added. Even for a qualitative or descriptive synthesis, some structured critical appraisal of included studies is necessary if the review is presented as systematic. Fifth, the unexplained “dependability rate of 85%” should be clarified or omitted. Sixth, the discussion and conclusion should be revised so that the strength of the claims aligns with the limitations of the evidence base, especially the single-database search, language restrictions, and the absence of quality appraisal.

**Constructive suggestions for improvement**

 The manuscript has a solid topical foundation and a useful descriptive overview of inclusion-related work in libraries. To improve it, the authors should begin by sharpening the rationale in the introduction and explicitly identifying the knowledge gap their review addresses. They should then report the review process with greater transparency, ideally by including clearer eligibility criteria, reviewer roles, data extraction procedures, and a reproducible thematic analysis workflow. 

**Overall recommendation in narrative form**

 This is a relevant and potentially valuable review on an important topic. The objectives are broadly clear, and the manuscript brings together literature that may be useful for researchers and practitioners interested in libraries, equity, and community engagement. However, the article currently has methodological weaknesses that limit reproducibility and weaken the evidentiary basis for some of its conclusions. A substantial revision is needed, particularly regarding the clarity of review design, transparency of screening, justification of exclusions, methodological appraisal of included studies, and alignment between the evidence presented and the claims made. Once these issues are addressed, the manuscript will be considerably stronger.

Are the rationale for, and objectives of, the Systematic Review clearly stated?

Partly

Is the statistical analysis and its interpretation appropriate?

Partly

If this is a Living Systematic Review, is the ‘living’ method appropriate and is the search schedule clearly defined and justified? (‘Living Systematic Review’ or a variation of this term should be included in the title.)

Not applicable

Are sufficient details of the methods and analysis provided to allow replication by others?

Partly

Are the conclusions drawn adequately supported by the results presented in the review?

Partly

Reviewer Expertise:

Ethnomusicology; Music Education; Cultural Heritage Studies; Qualitative Research Methodology; Arts and Humanities Research

I confirm that I have read this submission and believe that I have an appropriate level of expertise to state that I do not consider it to be of an acceptable scientific standard, for reasons outlined above.

# Response

**Published:** 2026-06-22

**Authors:** Ana Dolores Vargas Sánchez

**Affiliations:** Cundinamarca, Universidad de La Sabana, Chia, Cundinamarca, Colombia

We extend our sincere gratitude to the reviewer for their comprehensive assessment of our manuscript and for the constructive recommendations they offered. We have meticulously reviewed all comments and have made the necessary revisions to the manuscript. Detailed responses to each argument are provided below.

 The study's nature has been thoroughly elucidated throughout the manuscript, following a thorough evaluation. In particular, we are now offering a comprehensive account of the screening process, which encompasses the identification of the study, the appraisal of eligibility, and the final selection process. We have provided explicit inclusion and exclusion criteria in reproducible terms, clarified the responsibilities of reviewers during data extraction and screening, and elucidated the process by which discrepancies were resolved. In order to enhance transparency and facilitate reproducibility, these modifications were implemented. The Discussion and Conclusion sections have been revised. The manuscript has been revised to offer a more balanced interpretation of the findings and explicitly recognizes the limitations of the single-database search strategy, language restrictions, and the initial lack of a formal quality assessment. In order to guarantee that conclusions are in accordance with the available evidence, claims have been moderated.

# Reviewer report: Reviewer response for version 1

**DOI:** 10.5256/f1000research.193081.r463183

**Published:** 2026-03-10

**Authors:** Ida Fajar Priyanto

**Affiliations:** 1 Universitas Gadjah Mada, Yogyakarta, Special Region of Yogyakarta, Indonesia

Just a note:

 based on the practice of social inclusion, in average the librarians at public or rural libraries can only practice the social inclusion such as that in Indonesia. They cannot do research, but practice social inclusion well, including training for trainers as well. So, collaboration between librarians and faculty may help producing more research papers on social inclusion.

Are the rationale for, and objectives of, the Systematic Review clearly stated?

Yes

Is the statistical analysis and its interpretation appropriate?

Yes

If this is a Living Systematic Review, is the ‘living’ method appropriate and is the search schedule clearly defined and justified? (‘Living Systematic Review’ or a variation of this term should be included in the title.)

Yes

Are sufficient details of the methods and analysis provided to allow replication by others?

Yes

Are the conclusions drawn adequately supported by the results presented in the review?

Yes

Reviewer Expertise:

library social inclusion, Open Access, Informaion Behavior, library as space

I confirm that I have read this submission and believe that I have an appropriate level of expertise to confirm that it is of an acceptable scientific standard.
